# Breaking the biofilm barrier: herbomineral nanocomposites to control *Staphylococcus epidermidis* through oxidative response and biofilm disruption

**DOI:** 10.3389/fchem.2026.1917308

**Published:** 2026-09-21

**Authors:** Monisha Sabapathi, Ranjani Soundhararajan

**Affiliations:** School of Life Sciences, B. S. Abdur Rahman Crescent Institute of Science and Technology, Chennai, India

**Keywords:** confocal laser scanning microscopy, dental biofilm, green nanotechnology, herbomineral nanocomposites, reactive oxygen species, *S. epidermidis* pathogen

## Abstract

The growing prevalence of antibiotic resistance and biofilm-forming dental pathogens necessitates the development of alternative antimicrobial strategies. *Staphylococcus epidermidis* is an opportunistic pathogen associated with dental biofilms, oral microbial colonization, and implant-related infections, contributing to persistent infections and treatment challenges. In this study, a *herbomineral nanocomposite* (HMNCs) was fabricated using the traditional Siddha formulation Kalnar Parpam and silver nitrate, and its antibacterial and antibiofilm activities were evaluated against *S. epidermidis* isolates obtained from dental caries-associated samples. Physicochemical characterization supported the formation of HMNCs. UV–Vis spectroscopy showed a characteristic surface plasmon resonance peak around 400 nm, while FTIR analysis revealed functional groups associated with the mineral matrix and synthesized HMNCs. XRD confirmed the crystalline nature of the material, and FESEM showed clustered nanostructures with particle sizes ranging from 52.8 to 117.9 nm, with an average particle size of 70.15 nm. DLS showed a hydrodynamic diameter of 400.4 nm with a polydispersity index of 0.197, while the zeta potential was −3.7 mV. XPS confirmed the presence of silver on the HMNCs surface, and ICP-OES quantified the total silver content as 0.040 mg/g (40 μg/g). TGA showed an approximately 18% mass loss up to 800 °C, with substantial inorganic residue. HMNCs exhibited antibacterial activity, with MIC values ranging from 0.325 to 1.5 μg/mL and MBC values ranging from 0.325 to 3.125 μg/mL. The MBC/MIC ratios ranged from 1.00 to 2.15 based on the operational MBC endpoint used in this study. Resazurin assays demonstrated concentration-dependent suppression of bacterial metabolic activity. HMNCs exposure was associated with increased DCFDA fluorescence and MDA levels, together with changes in GSH, SOD, and CAT parameters and leakage of intracellular sugars, proteins and nucleic acids. Furthermore, HMNCs inhibited biofilm formation and reduced preformed biofilm biomass, as demonstrated by crystal violet staining, with complementary FESEM and CLSM analyses using the SE4 isolate. Overall, the findings provide preliminary *in vitro* evidence of the antibacterial and antibiofilm activity of HMNCs and support further investigation of this Siddha-inspired nanomaterial for potential oral healthcare applications.

## 1 Introduction

Dental infections are common chronic infectious illnesses caused by pathogenic organisms that cling to teeth and break down sugars to form acids, which eventually demineralize tooth structure. One of the biggest reservoirs of organisms is the mouth/oral cavity. The human mouth is a home that can accommodate between 700 and 1,000 different microbial species. The two most prevalent conditions affecting the oral cavity are caries and periodontal infections. In both conditions, the disease progression is brought about by inadequate oral hygiene and microcosm induced dental biofilms. Through damaged oral mucosa, pathogenic bacteria can enter the bloodstream and raise systemic antibody levels, which may trigger the risk of certain diseases including lung infections (COPD, pneumonia, etc.), bone and joint diseases, neurological complications, and cardiovascular conditions.

*Staphylococcus epidermidis* is a predominantly commensal Gram-positive bacterium that commonly colonizes the skin and mucosal surfaces but can act as an opportunistic pathogen under certain conditions (Chuchu et al., 2025). Although it is not considered a primary etiological agent of dental caries, *S. epidermidis* has been reported in oral and dental samples, including dental plaque, infected dental pulp, and inflammatory lesions (Devang Divakar et al., 2017). Its presence in such samples represents an association with the sampled oral environment and does not, by itself, establish a causal role in dental disease. The organism is also recognized for its ability to form biofilms and its involvement in healthcare-associated infections, particularly those associated with implanted medical devices (Sousa et al., 2014). Biofilm formation can contribute to bacterial persistence and reduced susceptibility to antimicrobial treatment. Therefore, investigating alternative antimicrobial and antibiofilm approaches against *S. epidermidis* isolates obtained from dental caries-associated samples may provide useful *in vitro* evidence regarding their potential application in oral healthcare (Chabi and Momtaz, 2019).

Green nanotechnology emerges as a transformative alternative by utilizing biological sources namely plant extracts, sea weeds, algae, bacteria, fungi, metabolites, microbial enzymes to enable ecofriendly synthesis. Conventional synthesis methods frequently rely on high energy consumption, toxic reducing agents and costlier facilities, which can leave hazardous residues on the resulting nanocomposites causing a critical concern for biomedical especially intraoral applications (Salem, 2023). By utilizing the secondary metabolites, phytocompounds, minerals from natural sources, simultaneous reduction and stabilization of nanocomposites can be achieved during the process of green synthesis. Developing biocompatible green based nanocomposites are safe for humans, animals, plants and environment. A nanocomposite is a highly engineered, multiphase solid material in which at least one component has dimensions less than 100 nm. These composites integrate highly reactive nanoscale component and structurally stable macroscopic phases thereby creating a hybrid system by embedding nanoscale phase in a macroscopic matrix. The structural matrix of the nanocomposite functions as a controlled delivery platform enabling sustained and prolonged release of drugs directly at the target location without subsequent treatment. Recent advances in nanotechnology have expanded the scope of nanomaterials beyond conventional antimicrobial applications toward multifunctional biomedical platforms integrating sensing, diagnosis, monitoring and therapeutic functions, highlighting the potential of next-generation nanomaterials for advanced healthcare applications (Kirubakaran, 2026). Nanocomposites often utilize metals and nanoscale reinforcements to improve mechanical properties including wear resistance and thermal stability. Owing to the antibacterial and biofilm eradicating properties, green nanocomposites are increasingly explored in biomedical, dental, dermal, food packaging, environmental applications and many more. Eco-friendly nanocomposites possess unique physicochemical properties allow them to penetrate deep into the microscopic niches of the oral cavity including dentinal tubules and periodontal pockets and execute its antimicrobial action (Ghodrati et al., 2025). Nanobased platforms not only offer a sustainable synthesis route; it also provides a therapeutic model where the bio-active compounds and natural minerals used during the synthesis process often enhance the ability of nanocomposites to combat dental pathogens (Silva et al., 2025). Antibacterial activity of green nanocomposites relies on multiple mechanisms including disruption of bacterial membrane and induction of lipid peroxidation rather than targeting specific enzymatic pathways, making it more difficult for the bacteria to become resistant. Consequently, this approach represents a frontier in precision dentistry, providing a robust, non-toxic, and ecologically responsible framework for developing nanocomposites to control persistent oral infections caused by microcosms.

Kalnar Parpam (KP) is an established mineral-based medicinal formulation used in the Siddha system of medicine under the broader AYUSH framework (AYUSH Standard Public Health, 2024.pdf, 2024). Siddha medicines are supported by established pharmacopoeial and formulary standards addressing aspects such as identity, quality, purity and strength. The recognized medicinal status and commercial availability of KP provide a rationale for its selection as the mineral component in the present study. In this work, commercially available KP was procured and used as received for the preparation of herbomineral nanocomposites (HMNCs). KP has a long-standing role in traditional Siddha practice and has been described for the management of various disorders, including dysuria, leucorrhea, urogenital inflammatory conditions, and disorders associated with excess internal body heat. Its traditional use in dental applications, particularly in conditions involving gingival inflammation, bleeding, and tooth-related disorders, further provides a basis for exploring its potential relevance to oral microbial environments. The mineral-rich nature and physicochemical characteristics of KP provide an interesting platform for integration with nanotechnology. Mineral-based materials may interact with microbial cells through their surface characteristics and chemical properties, while incorporation of silver can provide additional antimicrobial activity. The combination of a traditional herbomineral-based medicinal formulation with silver therefore offers an opportunity to develop a multifunctional nanomaterial with potential antibacterial and antibiofilm properties.

In the present study, KP was used as the mineral component for the fabrication of herbomineral nanocomposites (HMNCs), which were subsequently characterized using complementary physicochemical techniques. *Staphylococcus epidermidis* isolates from dental caries-associated samples were used to evaluate the *in vitro* antibacterial and antibiofilm activities of the fabricated HMNCs. The isolation of *S. epidermidis* from carious teeth was considered an association with the sampled dental environment and was not interpreted as evidence of a causal role in dental caries.

The antibacterial and antibiofilm activities of the HMNCs were evaluated against the *S. epidermidis* isolates obtained from dental caries-associated samples, along with oxidative stress-associated responses and cellular membrane integrity. These investigations were undertaken to assess the biological effects of the fabricated HMNCs and to explore their activity against biofilm-forming *S. epidermidis* isolates obtained from dental caries-associated samples. The findings provide preliminary *in vitro* evidence for the antimicrobial and antibiofilm potential of KP-derived HMNCs and establish a basis for further investigation of their applicability in oral healthcare-related nanomaterials.

## 2 Materials and methods

### 2.1 Preparation of herbomineral extract and physicochemical characterization

Kalnar Parpam (KP) was procured from IMCOPS, Chennai. 50 g of KP was weighed and mixed in 1,000 mL of water. The contents were allowed to mix well and boil for 300 s. The mixture was kept macerated for overnight period to achieve complete extraction of minerals. The extraction was centrifuged at 6,000 rpm for 10–15 min and filtered through Whatman filter paper (Ranjani et al., 2022). FESEM coupled with EDX was employed to analyze their morphology, size, elemental mapping, and elemental composition of KP. XRD was used to evaluate the crystalline nature, lattice arrangements, phase composition, and average crystallite size of KP. In order to analyze the elements in herbomineral formulation, KP was subjected to ICP OES analysis. 200 mg of the KP powder was dissolved in concentrated nitric acid (69%–70%, analytical reagent grade) was added (10 mL) and placed in an Anton Paar Multiwave Go Plus microwave digester. The mixture was heated to 190 degrees for 45 min. The sample was microwave-digested filtered to obtain a clear, colorless solution that was subsequently characterized using ICP-OES analysis.

### 2.2 Fabrication and characterization of herbomineral nanocomposites (HMNCs)

The fabrication of herbomineral nanocomposites (HMNCs) was achieved by mixing the herbomineral (KP) extract with 1 mM silver nitrate in a ratio of 4:1. The mixture was left undisturbed at room temperature for about 24–48 h under dark conditions. After visible colour change, the mixture was subjected to centrifugation at 7,000 rpm for 20 min (Sai Nivetha et al., 2020). The same process is achieved thrice to obtain maximum yield of HMNCs. Synthesized HMNCs were purified by repeated washing by adopting vortexing and centrifugation techniques. The pellets of HMNCs were finally purified with Milli Q water and dried in a hot air oven temperature at 80 °C. The dried HMNCs were sonicated to disperse them in 10% DMSO and subjected to high-speed homogenization to obtain well dispersed solution of HMNCs for further physico chemical and *in vitro* analysis. Fabricated HMNCs were analyzed using various characterization techniques to assess their size, shape, morphology, surface charge, particle distribution, topography, elemental composition and stability. The total silver content of HMNCs was determined by inductively coupled plasma–optical emission spectrometry (ICP-OES). Briefly, 200 mg of HMNCs was digested with 10 mL concentrated HNO_3_ using an Anton Paar Multiwave GO Plus microwave digestion system at 190 °C for 45 min. Following digestion, the solution was appropriately made up to obtain a 2000 ppm stock solution. The stock solution was subsequently diluted to 100 ppm, filtered to obtain a clear solution, and analyzed by ICP-OES. Three analytical measurements were performed for silver determination.

UV–visible spectroscopy was employed to characterize the optical and spectral properties of HMNCs. The identification of chemical groups that function as capping agents, stabilizers of molecular structures and functional groups residing on the HMNCs was analysed by FTIR (Xia et al., 2023). FESEM coupled with EDX was employed to analyze their morphology, size, elemental mapping and elemental composition of HMNCs. XRD was used to evaluate the crystalline nature, lattice arrangements, phase composition, and average crystallite size of synthesized HMNCs. The surface elemental composition and chemical states of the synthesized herbomineral nanocomposite (HMNCs) were analyzed using X-ray photoelectron spectroscopy (XPS) (Motas et al., 2021). A particle size analyzer using the Dynamic Light Scattering (DLS) technique in conjunction with a Zeta analyzer was used to examine the physicochemical behavior, hydrodynamic size, surface charge, polydispersity index, and colloidal stability of the HMNCs. HR-TEM was employed to determine the particle size, shape, morphology, and internal structural features of the fabricated HMNCs. SAED analysis was performed in conjunction with HR-TEM to evaluate the crystalline nature and phase purity of the HMNCs. The diffraction ring patterns obtained from SAED confirmed the crystalline structure of the nanocomposites and complemented the findings obtained from X-ray Diffraction (XRD) analysis. The crystallite size of the Kalnar Parpam and synthesized HMNCs was estimated using the Debye–Scherrer equation based on the full width at half maximum (FWHM) of the most intense diffraction peak obtained from the XRD pattern (Xia et al., 2023). Thermogravimetric Analysis (TGA) was carried out to evaluate the thermal stability and decomposition behavior of the HMNCs.

### 2.3 Isolation of *S. epidermidis* strains from infected dental samples

Dental swab samples were collected from carious teeth of systemically healthy patients with a high caries index (DMFT >4) attending Tagore Dental College and Hospital, Chennai. A total of 35 patients were included in the sample collection, and the samples were collected during the study period from October 2025 to August 2026. The study participants were within the age group of 18–70 years and were selected according to the predefined inclusion and exclusion criteria. Informed consent was obtained from the participants prior to sample collection. Sterile swabs were used to collect samples from the carious teeth and the samples were processed for bacterial isolation and subsequent molecular identification. The study protocol was reviewed by the Institutional Ethics Committee of Tagore Dental College and Hospital, Chennai, and was granted a Certificate of Exemption from Ethics Review (Ref. No. IEC/TDCH/133/2025; dated 05.01.2026). The proposal was exempted from full ethics review as an *in vitro* study conducted entirely on samples not involving human participants. Informed consent for dental swab collection was obtained as specified in the approved study protocol.

Dental swabs were collected and subjected to 10-fold serial dilution in peptone broth. Aliquot of the last dilution was streaked in mannitol salt agar and incubated for 24 h at 37 °C. Individual colonies were selected and subcultured to obtain pure bacterial cultures. A total of 61 bacterial isolates were recovered from the dental samples. Among these isolates, those identified as *S. epidermidis* were selected for the subsequent experiments. Eight *S. epidermidis* isolates (SE1–SE8) were finally used for the antibacterial, antibiofilm and mechanistic investigations.

Pure bacterial colonies obtained from the dental samples were submitted to Barcode BioSciences Pvt. Ltd., Bengaluru, India, for molecular identification. The 16S rRNA gene was amplified using the universal bacterial primers 27F and 1492R and approximately 1.5 kb of the 16S rRNA gene was sequenced using internal sequencing primers. The sequencing and sequence analysis were performed by the service provider. The generated sequences were analysed using Chromas Pro and aligned using Clustal X 2.0.

The 16S rRNA gene sequences obtained from isolates SE1–SE8 were compared with homologous sequences available in the NCBI GenBank database. Multiple sequence alignment was performed using MUSCLE implemented in MEGA. A Maximum Likelihood (ML) phylogenetic tree was constructed using the Tamura–Nei nucleotide substitution model with uniform rates among sites. Gaps and missing data were treated using all available sites. The Nearest-Neighbor-Interchange (NNI) method was used as the ML heuristic search method, with the initial tree generated automatically. The reliability of the inferred phylogenetic relationships was assessed using the Standard Bootstrap method with 1,000 bootstrap replicates. *Staphylococcus aureus* strain IraqYaAwSh22.6 (accession number LC891134) was used as the outgroup for tree rooting. Bootstrap values were displayed at the corresponding internal nodes.

Antimicrobial susceptibility testing of the isolated strains was performed using the Kirby–Bauer disk diffusion method. Briefly, bacterial cultures were adjusted to a turbidity equivalent to a 0.5 McFarland standard and uniformly inoculated onto Mueller–Hinton agar plates. Commercially available antibiotic disks obtained from HiMedia Laboratories, India, were placed aseptically on the inoculated agar surface using a disk dispenser. The antimicrobial agents tested were norfloxacin (10 µg), enrofloxacin (5 µg), penicillin-G (10 units), tetracycline (30 µg), gentamicin (10 µg), cefoperazone/sulbactam (75/30 µg), ceftriaxone (30 µg), and ampicillin (10 µg). The inoculated plates were incubated at 35 °C ± 2 °C for 24 h. Following incubation, the diameters of the inhibition zones were measured in millimetres. The disk diffusion procedure was performed with reference to the Clinical and Laboratory Standards Institute (CLSI) M02, 14th edition (2024). Applicable antimicrobial susceptibility interpretive criteria were consulted from CLSI M100, 36th edition (2026), where available. The measured inhibition-zone diameters were interpreted according to the applicable CLSI breakpoint criteria. A standardized CLSI ATCC/MTCC quality-control reference strain was not included during the original testing. Therefore, quality-control results from a reference strain were not available for the present study.

### 2.4 Assessment of anti-bacterial properties of HMNCs

Prior to biological evaluation, the synthesized HMNCs were dispersed in 10% DMSO by sonication followed by high-speed homogenization to obtain a uniform suspension. For LB ph value 7. This dispersion procedure was used consistently throughout all subsequent antimicrobial assays. The measured pH values were 7.30 for the NP suspension, 7.10 for LB broth, and 7.41 for the final LB broth–NP suspension, respectively.

#### 2.4.1 Microdilution turbidity assays

The minimum inhibitory concentration (MIC) of the synthesized HMNCs against the isolated *S. epidermidis* strains was determined using the broth microdilution method in 96-well microtiter plates. Luria-Bertani broth (LB) was used as the test medium. The bacterial inoculum was standardized to a 0.5 McFarland turbidity standard before inoculation. HMNCs were serially diluted in LB to obtain concentrations ranging from 100 to 0.325 μg/mL. A 2 µL aliquot of log-phase bacterial culture was added to the respective wells. The assay was performed in triplicate (n = 3). Wells containing LB and bacterial culture without HMNCs served as growth control, while LB without bacterial inoculum served as sterility control. Wells containing LB with the corresponding HMNCs concentrations but without bacterial inoculum were included as HMNCs-only controls to account for turbidity associated with the test material. Ampicillin (10 µg) was included as the antibiotic control. The plates were incubated at 37 °C for 24 h. Following incubation, bacterial growth was assessed visually based on turbidity. The MIC was recorded as the lowest HMNCs concentration at which no visible bacterial growth was observed compared with the untreated bacterial growth control.

The Minimum Bactericidal Concentration (MBC) of HMNCs was determined following the MIC assay. Drop plate assay was carried out by inoculating 1 μL from each well of the microtiter plates into LB agar plate and incubated at 37 °C for 24 h (Aflakian and Hashemitabar, 2025). Following incubation, the agar plates were examined visually for the presence or absence of bacterial growth. The concentration of HMNCs at which no visible growth was recorded as MBC. The assay was performed in triplicate (n = 3). Quantitative colony-forming-unit enumeration was not performed; therefore, the MBC values were interpreted as operational endpoints based on absence of visually detectable growth rather than as quantitatively determined bactericidal concentrations based on a predefined reduction in viable bacterial counts.

#### 2.4.2 Bacterial growth kinetics

The time-dependent effect of HMNCs treatment on the growth of *S. epidermidis* strains was evaluated by measuring the optical density at 600 nm (OD600). Bacterial cultures were treated with HMNCs and ampicillin (10 µg) and OD at 600 nm were recorded at 12 h intervals for up to 48 h. Untreated bacterial cultures were maintained as growth controls. Particle-only blanks containing HMNCs without bacterial cells were included at the corresponding concentrations to account for the background optical contribution of the HMNCs. The changes in OD600 over time were used to evaluate the growth-inhibitory effect of HMNCs treatment compared with the untreated and antibiotic-treated cultures (Khalifa et al., 2025).

#### 2.4.3 Resazurin-based bacterial viability assay

Resazurin-based bacterial viability assay was performed similarly to that of microdilution turbidity assays but in a dark 96 well plate (Travnickova et al., 2019). Plates were prepared with blank, positive control, standard antibiotics and HMNCs treatment in respective strains. Dark plates were incubated for 24 h at 37 °C. After incubation 20 µL of the resazurin working solution was added, the second incubation was carried out for 2–4 h at 37 °C in dark. Fluorescence with excitation Ex = 530–570 nm and emission Em = 590–620 nm was measured using a microtiter plate reader. Absorbance was recorded for every half hour till 2 h and the difference between the two readings represented the delta fluorescence for the reduced resazurin dye.

### 2.5 *In vitro* investigations to explore the antibacterial mechanism of HMNCs in isolated dental pathogens

To investigate the antibacterial mechanism of HMNCs against isolated dental pathogens, all bacterial strains were revived and standardized to a concentration of 10^6^ CFU/mL. The bacterial cultures were treated with sub-MIC concentrations of HMNCs and ampicillin (10 µg), while untreated cultures were served as controls. All experimental tubes were incubated at 37 °C for 24 h in a bacteriological incubator.

#### 2.5.1 Assessment of oxidative stress

##### 2.5.1.1 Measurement of malondialdehyde (MDA)

The thiobarbituric acid (TBA) assay was used to quantify the lipid peroxidation in bacterial cells. Collected cell pellets of treatment and controls were suspended in SDS (10%) and mixed with equal volume of TBA (freshly prepared). Contents were heated at 95 °C for 60 min. The contents were gradually cooled and centrifuged. The absorbance of the resulting supernatant was recorded at 530 and 600 nm (Harine et al., 2025). The measured MDA levels were used as an indicator of membrane lipid oxidation and oxidative stress in the bacterial cells.

##### 2.5.1.2 Determination of reduced glutathione (GSH)

The cell pellets of control and treatment groups were washed with PBS buffer. Pellets were lysed to release the intracellular contents. The GSH level was estimated by using Ellman’s reagent (Harine et al., 2025). The absorbance was measured at 412 nm. The absorbance measurements enabled for the evaluation of changes in the cellular antioxidant capacity in response to treatment with HMNCs and provide quantitative information on the GSH levels in the bacterial cells with and without treatment.

##### 2.5.1.3 Catalase (CAT) activity

Catalase activity was evaluated by observing the decomposition of hydrogen peroxide. The cell pellets of control and treatment groups were washed with PBS buffer and resuspended in buffer (Harine et al., 2025). The initial absorbance of all the control and treated samples were measured at 240 nm. Later, hydrogen peroxide was then added to all the samples to initiate the reaction. The decrease in absorbance at 240 nm was monitored and used to calculate catalase activity. Measurement represents the rate at which catalase breaks down hydrogen peroxide.

##### 2.5.1.4 Superoxide dismutase (SOD) activity

Superoxide Dismutase (SOD) activity was determined using nitroblue tetrazolium (NBT) reduction method. Bacterial pellets of control and treatment groups were washed and suspended in PBS. The reaction mixture comprised of phosphate buffer (50 mM, pH 7.0), EDTA (0.1 mM), methionine (13 mM), NBT (63 μM), riboflavin (1.3 μM), and 0.1 mL of the bacterial suspension. Reaction mixture was distributed into a 96-well plate and incubated in dark for 15 min and absorbance was measured at 560 nm (Harine et al., 2025). SOD activity was determined from the inhibition of NBT reduction by superoxide radicals generated during the reaction.

##### 2.5.1.5 Intracellular reactive oxygen species (ROS) assay

Intracellular ROS generation was measured using 2′,7′-dichlorodihydrofluorescein diacetate (DCFH-DA) (Eruslanov and Kusmartsev, 2010). Bacterial cultures were exposed to HMNCs at their respective sub-MIC values and incubated at 37 °C for 24 h. The cells were then collected by centrifugation at 10,000 rpm for 30 min at 4 °C and washed with PBS. Cell pellets from control, ampicillin-treated, and HMNCs-treated groups were incubated with 10 μM DCFH-DA at 37 °C for 45 min. Fluorescence intensity was measured using a microplate reader at excitation and emission wavelengths of 485 nm and 519 nm, respectively. ROS production was expressed as the percentage fluorescence relative to the untreated control.

#### 2.5.2 Assessment of cellular membrane integrity by measuring the leakage of intracellular components

After completion of incubation period, the bacterial cultures were subjected to centrifuged (4 °C) at 10,000 rpm for 30 min. The supernatants and cell pellets of the respective groups were carefully separated and stored at −20 °C for further analyses. The integrity of the bacterial cell membrane was assessed by quantifying the leakage of intracellular constituents including proteins, sugars, and nucleic acids.

After careful processing, the supernatant of HMNCs treatment, ampicillin treatment and respective controls groups containing leaked cellular components (proteins, sugars, and nucleic acids) was analyzed by using the Bradford method and the Anthrone method respectively for measuring total proteins and sugars (Harine et al., 2025). The extracellular nucleic acid was estimated by measuring the absorbance of the supernatant from control and treated cultures at 260 nm (Leena Panigrahi et al., 2023).

The measurement of leakage of proteins, sugars, nucleic acids provide detail insight about the membrane damage induced by HMNCs treatment.

### 2.6 Evaluation of antibiofilm activity of HMNCs in *Staphylococcus epidermidis*

#### 2.6.1 Biofilm prevention and disruption of preformed biofilms using crystal violet staining

Biofilm inhibition (prevention) assay by crystal violet staining was performed in 96-well plates using a microdilution-based assay. The experimental groups included sterile LB broth as the blank control, bacterial culture without treatment as the untreated control, bacterial culture treated with ampicillin (10 μg) as the antibiotic-treated group, bacterial culture treated with serially diluted HMNCs (100–0.325 μg/mL) as the HMNCs-treated groups and serially diluted HMNCs (100–0.325 μg/mL) in broth without bacterial culture as HMNCs-only controls. Plates were incubated statically at 37 °C for 48 h to allow biofilm formation in the presence of the respective treatments (Kumar et al., 2026).

The preformed-biofilm disruption assay was performed using a similar 96-well plate procedure (Amankwah et al., 2021). In this model, bacterial cultures were first incubated without treatment for 48 h at 37 °C under static conditions to allow the biofilms to mature. Following biofilm maturation, the spent medium was removed and the preformed biofilms were treated with ampicillin (10 μg) and serially diluted HMNCs (100–0.325 μg/mL). The plates were then additionally incubated at 37 °C for 24 h under the same conditions to evaluate the effect of the treatments on preformed biofilm biomass. HMNCs-only wells without bacterial culture were included as background controls.

After completion of the respective incubation period, the culture medium was discarded and the wells were washed thoroughly to remove planktonic and loosely attached cells. The plates were allowed to dry completely, followed by addition of 100 μL of 0.1% crystal violet solution to each well. After 30 min of staining, the wells were washed and 30% glacial acetic acid was added to distain the crystal violet. The absorbance was measured at 595 nm to assess the attached biofilm biomass to determine the effects of HMNCs on biofilm formation and disruption of preformed biofilm biomass in comparison with the respective controls.

#### 2.6.2 FESEM analysis to study biofilm architecture in inhibition (prevention) and disruption model

Based on the antibiotic profile, *S. epidermidis* strain 4 (SE 4) was resistant to Penicillin–G, Tetracycline, Gentamicin, Cefoperazone/Sulbactam, Ceftriaxone was selected to study the effect of HMNCs on biofilm architecture using Field Emission Scanning Electron Microscopy (FESEM) in both prevention and disruption model.

In biofilm prevention model, sterile glass coverslips were placed in 6-well plates containing LB broth. Each well were inoculated with SE 4 strain (Aiman Khan et al., 2025). Sub MIC concentration of HMNCs were added simultaneously to the wells and the plates were incubated at 37 °C for 48 h to allow biofilm formation in the presence of HMNCs treatment.

In preformed biofilm disruption model, sterile glass coverslips were placed in 6-well plates containing LB broth. Each well was inoculated with SE 4. Plates were incubated at 37 °C for 48 h to allow formation of mature biofilm. After maturation of biofilm, used media was carefully aspirated and replenished with fresh media containing sub-MIC concentration of HMNCs. Plates were additionally incubated at 37 °C for 24 h to evaluate the biofilm disruption ability of HMNCs in preformed biofilms.

After completion of incubation in both the models, coverslips were gently washed with phosphate-buffered saline (PBS) (three times) to remove loosely attached and planktonic cells. The adhered biofilms were fixed by using 2.5% glutaraldehyde for 4 h at room temperature. After fixation, the coverslips were rinsed three times with PBS and subjected to a graded ethanol dehydration series (30%, 50%, 70%, 90%, and 100%). The dehydrated samples were air-dried and mounted on FESEM (Aiman Khan et al., 2025). The surface morphology and structural architecture of the biofilms were then examined using a Field Emission Scanning Electron Microscope (FESEM) to assess changes in biofilm morphology, density and structural integrity following HMNCs treatment.

#### 2.6.3 CLSM analysis to study biofilm-associated viability and spatial coverage

CLSM analysis was performed on *S. epidermidis* (SE4) biofilms following acridine orange/ethidium bromide (AO/EtBr) staining. SE4 was selected based on its antibiotic-resistance profile. Biofilms were established under the prevention and preformed biofilm disruption conditions described above. In the biofilm prevention model, bacterial cultures were grown on sterile glass coverslips in the presence of HMNCs and incubated at 37 °C for 48 h. In the preformed biofilm disruption model, mature biofilms were first established on coverslips by incubating the bacterial cultures for 48 h. The mature biofilms were then treated with HMNCs and further incubated for 24 h (Kumar Tiwari et al., 2019).

Following incubation, the coverslips were gently washed with PBS to remove non-adherent cells and other particulate materials. The biofilms were stained with acridine orange (AO) and ethidium bromide (EtBr) and incubated in the dark for 15 min. Excess stain was removed by washing with PBS and the stained biofilms were examined using an Olympus FLUOVIEW confocal laser scanning microscope equipped with a 40× objective (LUCPLFLN 40X). AO fluorescence was used to visualize predominantly viable cells, whereas EtBr fluorescence represented membrane-compromised cells.

Images were acquired at a resolution of 2048 × 2048 pixels, with green and red fluorescence signals collected over 500–540 nm and 570–670 nm, respectively. The same imaging settings were maintained for the control and HMNCs-treated groups. Three representative fields were acquired for each sample. Images were processed using ImageJ software (version 1.54g). The biofilm-covered area and relative proportions of AO-positive (green) and EtBr-positive (red) fluorescence regions were quantified using the same image-processing and thresholding criteria across the experimental groups. The percentage reduction in biofilm-covered area was calculated relative to the untreated control.

The CLSM analysis was used as a complementary fluorescence-based assessment of biofilm-associated viability and spatial coverage. As the CLSM imaging was performed as a single experimental acquisition with three image fields obtained per sample, these image fields were treated as technical/image-level measurements rather than independent biological replicates. Therefore, the CLSM findings were not considered a direct determination of viable biofilm cell counts or complete biofilm disruption.

### 2.7 Statistical analysis

Assays were performed in triplicate (n = 3). Biological replicates are tests on independent samples, and the experimental results were analyzed using two-way ANOVA, with descriptive statistics including mean and standard deviation. Tukey’s *post hoc* test was performed, and the significance of all statistical tests was predetermined at p < 0.05 (*), p < 0.01 (**), p < 0.001 (***).

## 3 Results

### 3.1 Analysis of Kalnar Parpam extract

ICP-OES analysis of Kalnar Parpam extract indicates the presence of phosphorus, sulfur, iron, magnesium, calcium, potassium, sodium. Sulfur was identified as the predominant element with a concentration of 28.781 ppm, followed by calcium at 17.283 ppm. Trace levels of potassium (0.233 ppm), iron (0.094 ppm), sodium (0.064 ppm), magnesium (0.007 ppm), and phosphorus (0.005 ppm) were detected with relative standard deviation. Elemental analysis of KP confirms the presence of both major and trace elements. FESEM analysis of KP revealed an irregular and heterogeneous surface morphology with particle-like structures of varying dimensions (Figure 1A). The particle-size analysis of selected particle-like objects showed a mean size of 122.6 nm (Figure 1B). EDX analysis demonstrated the presence of carbon, oxygen, sulfur, and calcium, with oxygen (38.99 wt%) and calcium (35.34 wt%) representing the major detected elements, followed by sulfur (23.86 wt%) and carbon (1.81 wt%) (Figures 1C,D). Elemental mapping further demonstrated the spatial distribution of oxygen, silicon, sulfur, and calcium across the analyzed region (Figures 1E–I). These findings demonstrate the diverse elemental composition of KP and support its mineral-rich nature as the starting material for the subsequent fabrication of HMNCs.

**FIGURE 1:**
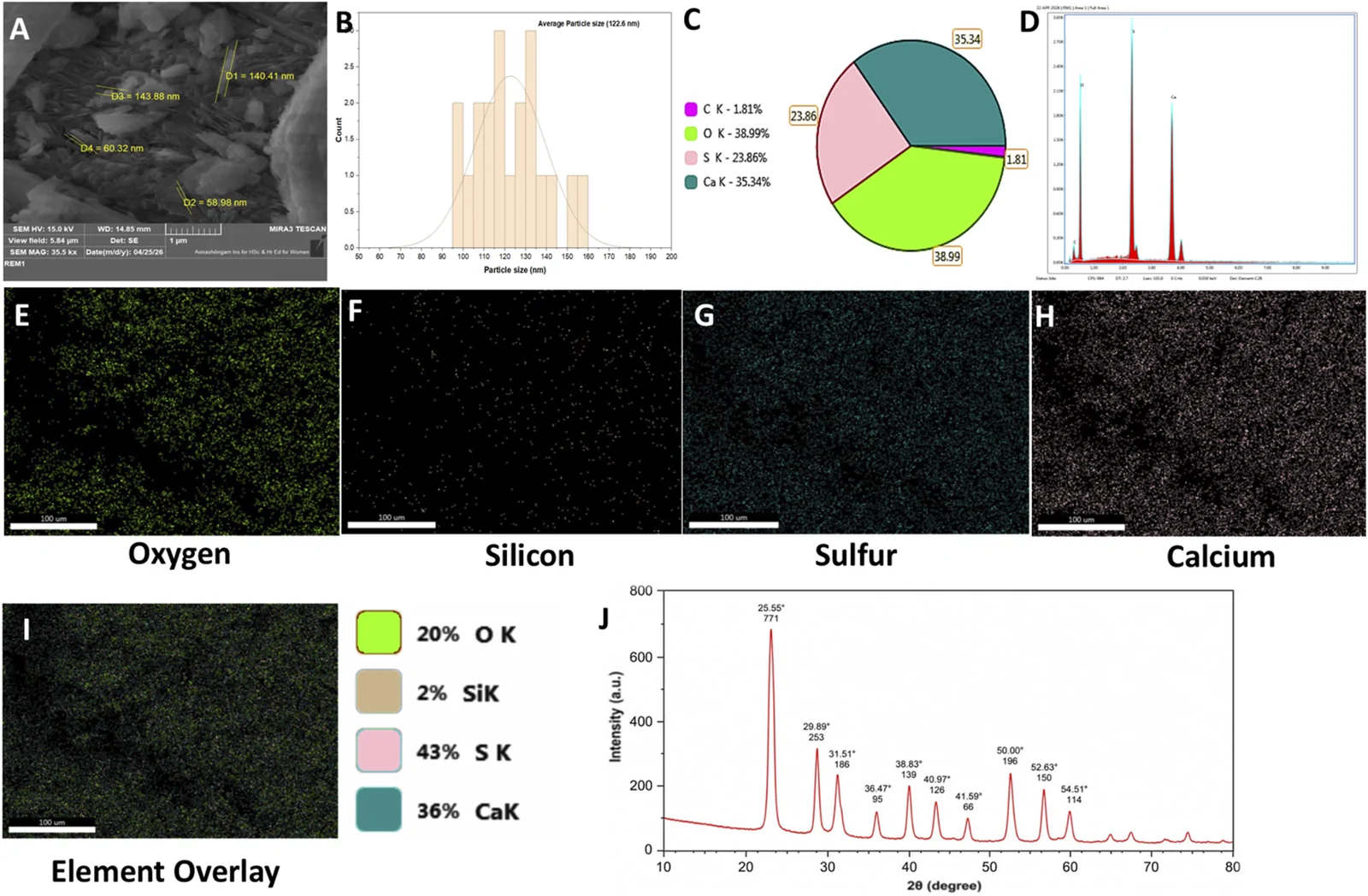
FESEM, particle-size distribution, EDX elemental composition and elemental mapping of Kalnar Parpam (KP): **(A)** FESEM micrograph showing the morphology and representative particle-size measurements; **(B)** Particle-size distribution with a mean particle size of 122.6 nm; **(C)** Quantitative EDX elemental composition showing carbon, oxygen, sulfur, and calcium; **(D)** Corresponding EDX spectrum; **(E–H)** elemental maps showing the spatial distribution of oxygen, silicon, sulfur, and calcium, respectively; and **(I)** composite elemental overlay map; **(J)** XRD analysis of KP depicting the crystalline phases.

The crystalline characteristics of KP were investigated using powder X-ray diffraction (XRD) and the resulting diffractogram exhibited several well-defined reflections, confirming the predominantly crystalline nature of the material (Figure 1J). The major diffraction peak was observed at 2θ = 25.55°, with a maximum intensity of 771 a.u. Additional prominent reflections were detected at 29.89° (253 a.u.), 31.51° (186 a.u.), 36.47° (95 a.u.), 38.83° (139 a.u.), 40.97° (126 a.u.), 41.59° (66 a.u.), 50.00° (196 a.u.), 52.63° (150 a.u.), and 54.51° (114 a.u.). The presence of multiple sharp reflections indicates that KP possesses an ordered crystalline mineral structure rather than a predominantly amorphous matrix. The diffraction pattern also demonstrates the multiphase nature of KP, which is consistent with its mineral-rich composition. The presence of multiple reflections across the 2θ range of approximately 25°–55° indicates contributions from mineral phases containing silicate and calcium, magnesium-associated components (Figure 1K).

### 3.2 Fabrication and characterization of HMNCs

The synthesis of KP based herbomineral nanocomposites (HMNCs) was carried out by adding silver nitrate solution into the KP extract and left for reduction for 24 h. A distinct colour change from milky white suspension to dark brown dispersion was observed (Figure 2A). The prominent change in colour was the signature of nanoparticles synthesis indicating the reduction of silver ions. ICP-OES analysis of the synthesized HMNCs demonstrated the presence of phosphorus, sulfur, iron, magnesium, calcium, potassium and sodium, along with silver. Calcium was identified as the predominant element with a concentration of 77.698 ppm, followed by sulfur at 4.706 ppm. Potassium (2.105 ppm), sodium (4.284 ppm), magnesium (0.797 ppm), iron (0.419 ppm) and phosphorus (0.118 ppm) were detected at comparatively lower concentrations, while silver was detected at 0.004 ppm in the analyzed solution. The three analytical measurements for silver were 0.004, 0.004, and 0.005 ppm, with an RSD of 5.7%. These findings demonstrate that the synthesized HMNCs contain both major and trace mineral elements, together with silver.

**FIGURE 2:**
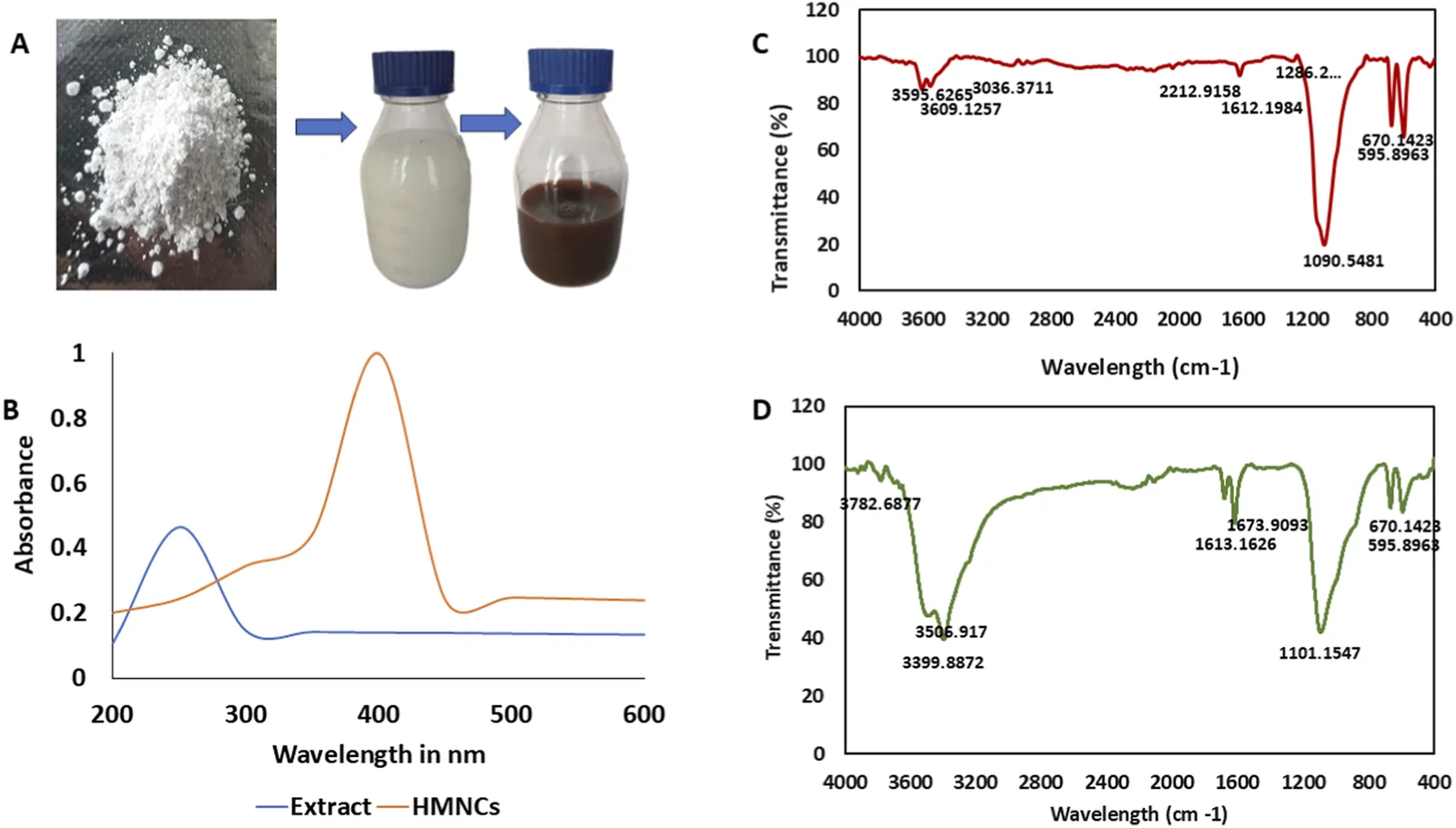
Synthesis and spectroscopic characterization of herbomineral nanocomposites (HMNCs): **(A)** Colour change observed during the fabrication of HMNCs indicating the reduction of silver ions; **(B)** UV–Visible spectrum of Kalnar Parpam extract and HMNCs depicting the characteristic SPR peak; **(C)** Fourier transform infrared spectrum of Kalnar Parpam extract indicating the functional groups involved in the synthesis of HMNCs; **(D)** Fourier transform infrared spectrum of synthesized HMNCs indicating the presence of functional groups that are inherited from Kalnar Parpam extract.

The synthesis of herbomineral nanocomposites (HMNCs) was further investigated by UV-Vis spectroscopy. The KP extract showed a UV-Vis s spectral peak at 250 nm (Figure 2B). The addition of silver nitrate followed by reduction (visible colour change), synthesized HMNCs showed a distinct spectral peak around 400 nm. The appearance of the absorption band at 400 nm, complemented by the reduction in intensity of the peak at 400 nm corresponding to the KP extract, indicates a significant change in the optical properties of the system after synthesis of HMNCs.

The pH of the HMNCs suspension, LB broth, and the final LB broth–HMNCs suspension was measured using a calibrated digital pH meter. The measured pH values were 7.30 for the HMNCs suspension, 7.10 for LB broth, and 7.41 for the final LB broth with HMNCs suspension. The pH measurements were performed under the same experimental conditions used for the antibacterial assay.

The FTIR spectrum of KP exhibited several characteristic absorption bands corresponding to mineral-derived functional groups (Figure 2C). A broad absorption band observed in the region of 3,600–3,550 cm^-1^ corresponds to O–H stretching vibrations, indicating the presence of structural hydroxyl groups and adsorbed moisture. A weak absorption band near 1,620 cm^-1^ corresponded to H–O–H bending vibrations of bound water molecules that may be trapped inside the parpam. A strong and prominent absorption band was observed at approximately 1,100–1,030 cm^-1^, which is characteristic of Si–O–Si and Si–O stretching vibrations. The presence of additional absorption around 670 cm^-1^ and 596 cm^-1^ corresponds to Si–O bending and metal–oxygen stretching vibrations, respectively (Table 1). FTIR spectrum of herbomineral formulation indicates the presence of silicate-rich minerals with inorganic constituents such as calcium and magnesium.

**TABLE 1:** FTIR spectrum of Kalnar Parpam

Peak (cm^-1^)	Functional group	Vibrational mode	Possible mineral/Compound assignment	Significance in Kalnar Parpam
3,609	O–H	Stretching	Structural hydroxyl groups	Indicates bound hydroxyl/moisture
3,596	O–H	Stretching	Hydroxyl-containing minerals	Surface hydroxyl functionality
1,612	C=C/H–O–H	Stretching/Bending	Adsorbed water or mineral-associated groups	Confirms moisture/mineral interactions
1,286	C–O	Stretching	Carbonate/silicate-associated groups	Indicates oxygen-containing mineral framework
1,090	Si–O–Si/C–O	Stretching	Silicate phase	Major mineral matrix
670	Si–O/Metal–O	Bending	Silicate minerals	Confirms mineral lattice
596	Ca–O/Mg–O/Metal–O	Stretching	Calcium and magnesium oxides	Supports ICP-OES findings

The FTIR spectrum of the fabricated HMNCs exhibited characteristic absorption bands corresponding to mineral-derived functional groups (Figure 2D). A broad absorption peak in the region of 3,560–3,400 cm^-1^ corresponds to O–H stretching vibrations, indicating hydroxyl groups which may be associated with phytochemicals and minerals. A definite absorption band by 1,630 cm^-1^ corresponded to O–H bending and/or carbonyl-associated vibrations, suggesting the presence of bioactive compounds involved in the synthesis and stabilization of the nanocomposite. A strong absorption band at approximately 1,100–1,030 cm^-1^, can be assigned to Si–O–Si and Si–O stretching vibrations which is the characteristic of silicate rich minerals. Additional peaks around 670 cm^-1^ and 590 cm^-1^ corresponds to Si–O bending and metal–oxygen stretching vibrations, respectively (Table 2). These spectral data indicate the existence of silicate-rich mineral constituents and metal-containing phases within the fabricated HMNCs.

**TABLE 2:** FTIR spectrum of herbomineral nanocomposites (HMNCs).

Wavenumber (cm^-1^)	Functional group/Bond	Vibrational mode	Possible assignment	Significance in HMNCs
3,560–3,400	O–H	Stretching	Hydroxyl groups from phytochemicals and surface-bound moisture	Indicates capping/stabilizing biomolecules
∼1,630	O–H/C=O	Bending/Stretching	Bound water and carbonyl-containing phytoconstituents	Suggests involvement of plant metabolites in synthesis
1,100–1,030	Si–O–Si/Si–O	Stretching	Silicate mineral framework	Confirms retention of Kalnar Parpam mineral matrix
∼950–900	Si–OH	Stretching	Surface silanol groups	Indicates active mineral surface sites
∼670	Si–O	Bending	Silicate minerals	Supports mineral-derived composition
∼590	Ca–O/Mg–O/Ag–O	Stretching	Metal–oxygen lattice vibrations	Indicates mineral and silver-associated phases
520–550	Metal–O	Stretching	Calcium, magnesium and silver-containing phases	Confirms inorganic nanocomposite framework

The fine powder of HMNCs was subjected to field-emission scanning electron microscopy (FESEM) to analyse the surface morphology and particle size of the HMNCs (Figure 3A). The FESEM micrograph displayed the distribution and arrangement of compactly packed nanoparticles with polymorphic morphology, along with aggregation/agglomeration. Based on the observed morphology, the nanoparticles within the nanocomposite matrix appeared heterogeneously clustered throughout the analysed surface area. In addition to the polymorphic morphology, elongated/needle-like crystalline structures were also observed within the nanocomposite matrix (Figure 3A). Particle-size analysis of 20 selected particles from the FESEM image showed particle sizes ranging from 52.8 to 117.9 nm, with an average particle size of 70.15 nm (Figure 3B). These observations indicate a rough and heterogeneous morphology with clustered/agglomerated particles, reflecting the complex particulate architecture of the HMNCs.

**FIGURE 3:**
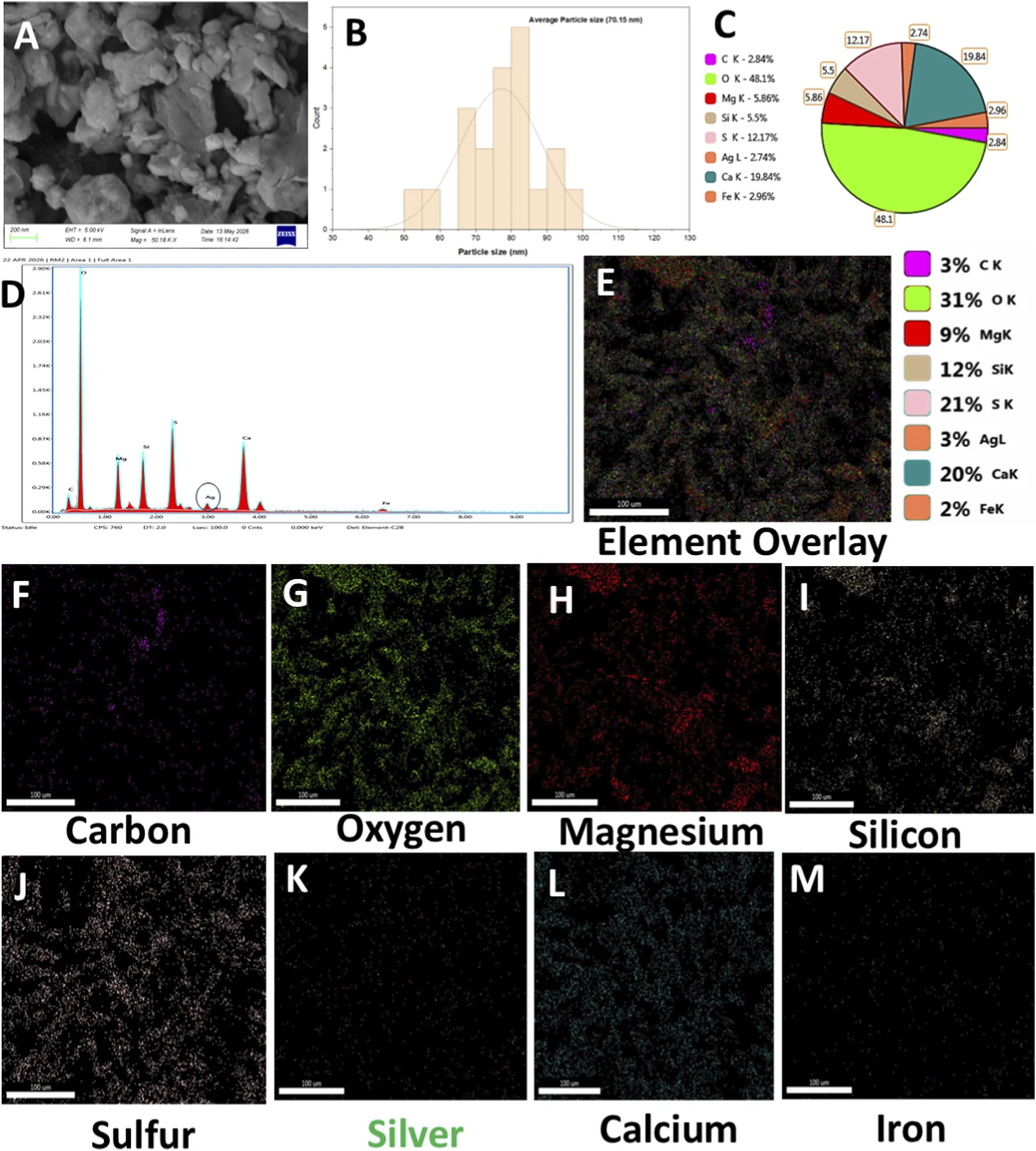
FESEM, particle size distribution, EDX spectrum, and elemental mapping of HMNCs: **(A)** FESEM image showing the surface morphology and nanoscale particulate structure of the synthesized HMNCs; **(B)** Particle-size distribution obtained from measurements of selected particles, showing an average particle size of 70.15 nm; **(C)** Quantitative EDX elemental composition of the HMNCs, showing the presence of C, O, Mg, Si, S, Ag, Ca, and Fe; **(D)** Representative EDX spectrum confirming the presence of the detected elements, including Ag; **(E)** EDX elemental overlay showing the spatial distribution of the detected elements within the analyzed region; **(F–M)** Individual elemental maps showing the distribution of Ca, O, Mg, Si, S, Ag, Ca and Fe, respectively.

The elemental composition of the HMNCs was determined using energy-dispersive X-ray spectroscopy (EDX) coupled with FESEM (Figures 3C,D). The EDX analysis revealed the presence of carbon, oxygen, magnesium, silicon, sulfur, silver, calcium, and iron, with weight percentages of 2.84%, 48.10%, 5.86%, 5.50%, 12.17%, 2.74%, 19.84%, and 2.96%, respectively. The corresponding atomic percentages were 5.10%, 64.90%, 5.20%, 4.22%, 8.20%, 0.55%, 10.69%, and 1.14%, respectively (Figure 3C). Oxygen was the most abundant element, followed by calcium and sulfur, while magnesium, silicon, iron, carbon, and silver were detected in lower proportions. The representative EDX spectrum showed characteristic signals corresponding to the detected elements, including a silver signal around 3 keV (Figure 3D), supporting the presence of silver in the synthesized HMNCs.

Elemental mapping further demonstrated the spatial distribution of carbon, oxygen, magnesium, silicon, sulfur, silver, calcium, and iron throughout the analysed region (Figures 3E–M). The elemental overlay and individual maps indicated the presence and distribution of these elements within the analysed HMNCs region (Figures 3E–M). The detection of both the mineral-associated elements and silver supports the presence of silver-containing material within the synthesized HMNCs. However, EDX analysis alone cannot establish the oxidation state of silver or confirm whether silver is present as metallic Ag or Ag^+^-containing species; therefore, the chemical state of silver was further evaluated using XPS.

The crystalline makeup of the fabricated herbomineral nanocomposites (HMNCs) was analysed by using X-ray diffraction (XRD) analysis (Figure 4A). The diffraction pattern of HMNCs showed several peaks at 2θ values of 20.99°, 21.24°, 23.69°, 29.36°, 29.64°, 31.60°, 33.61°, 36.91°, 41.08°, 43.88°, 48.27°, 50.55°, 56.96°, 57.14°, 68.91°, and 74.31°. The resemblance of multiple peaks is the representation of multiple crystalline phases within the synthesized nanocomposite. Diffraction peak at 29.36° displayed highest intensity, indicating the predominance of a major crystalline phase in the material. Based on the calculation using the Debye–Scherrer equation the average crystallite size from the intense diffraction peak (2θ = 29.36°, FWHM = 0.0836°) was approximately 98–100 nm, indicating the formation of nanocrystalline domains within the fabricated HMNCs.

**FIGURE 4:**
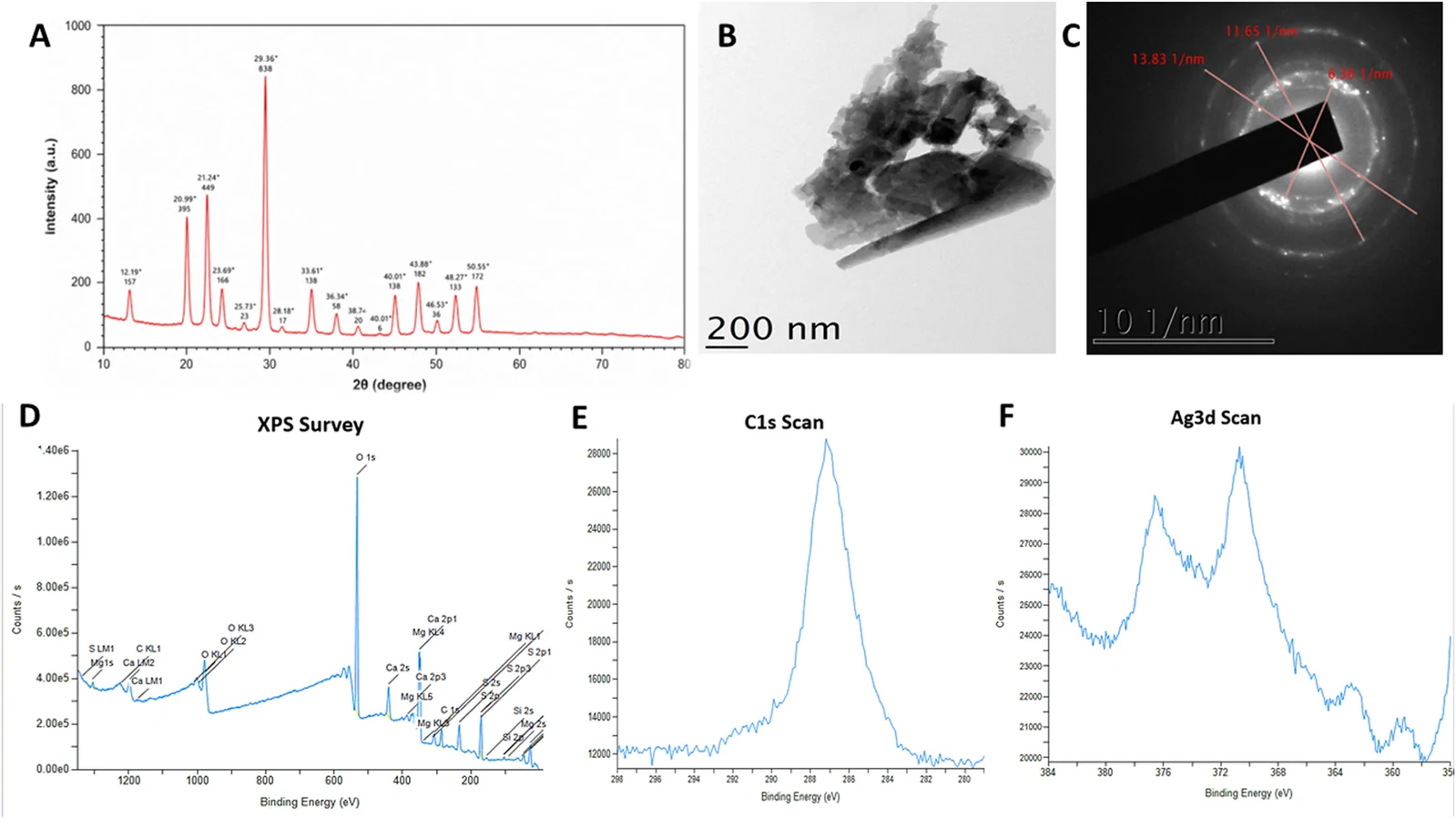
Morphological, elemental, structural and surface chemical characterization of HMNCs: **(A)** XRD pattern of HMNCs showing the characteristic diffraction peaks corresponding to the crystalline phases; **(B)** HRTEM image depicting the nanoscale morphology of HMNCs; **(C)** SAED pattern demonstrating the crystalline nature of HMNCs; **(D)** XPS survey spectrum showing the elemental composition of the HMNCs surface; **(E)** High-resolution C 1 s spectrum used for charge correction with the reference binding energy of 284.8 eV; and **(F)** High-resolution Ag 3d spectrum showing the characteristic Ag 3d doublet, supporting the presence of silver on the HMNCs surface.

HMNCs were subjected to HRTEM analysis to investigate the morphology and internal structure of the nanocomposite. The representative HRTEM micrographic image with 200 nm scale (Figure 4B) revealed irregular shaped plate-like and flake-like nanostructures aggregated into larger clusters of nanocomposites. The particles exhibit a layered structure with angular edges and diverse dimensions in nanometer in size. The composite matrix was observed as densely packed with interconnected aggregates of heterogenous particles. HRTEM analysis confirms the successful fabrication of herbomineral nanocomposite in nanoscale with unique structural organizations.

The crystallinity of the HMNCs was further investigated using selected area electron diffraction (SAED) analysis (Figure 4C). Based on the SAED analysis, it was observed as series of concentric diffraction rings with distinct bright spots indicating the existence of crystallinity within nanocomposite. Upon examining the ring patterns of HMNCs, it was confirmed the polycrystalline nature with multiple crystallopgraphic orientations. The measured reciprocal lattice spacings obtained from the diffraction rings were 13.83, 11.65, and 6.36 nm^-1^, corresponding to interplanar spacings (d-values) of approximately 0.072, 0.086, and 0.157 nm, respectively.

The selected-area electron diffraction (SAED) pattern exhibited concentric diffraction rings, indicating the crystalline nature of the synthesized nanocomposite. The diffraction features were evaluated in reciprocal space, and the corresponding interplanar spacings were calculated using the relationship (d = 1/g), where (g) represents the reciprocal lattice spacing (nm^-1^) and (d) represents the interplanar spacing (nm). The measured reciprocal-space values of 13.83, 11.65, and 6.36 nm^-1^ correspond to calculated d-spacings of approximately 0.0723, 0.0858, and 0.1572 nm, respectively. These values are reported with their appropriate units and are used for comparison with the expected crystallographic reflections of the constituent phases.

The surface chemical composition of the prepared HMNCs was investigated using X-ray photoelectron spectroscopy (XPS). The XPS survey spectrum (Figure 4D) revealed the presence of C, O, Si, Ca, Mg, and S as the major surface elements. The high-resolution C 1 s spectrum (Figure 4E) displayed a broad and asymmetric envelope extending approximately from 280 to 298 eV, with the major intensity observed in the 287–288 eV region. The high-resolution Ag 3d spectrum (Figure 4F) exhibited two prominent components corresponding to the Ag 3d_5_/_2_ and Ag 3d_3_/_2_ spin–orbit doublet, with an apparent separation of approximately 6.0 eV. The observed Ag 3d signals further support the presence of silver on the HMNCs surface. Taken together, the XPS results indicate a complex, oxygen-rich Ca–Mg–Si-containing inorganic surface matrix with detectable silver.

The particle size distribution of the synthesized herbomineral nanocomposites (HMNCs) was determined by dynamic light scattering (DLS). The hydrodynamic diameter of 400.4 nm with a polydispersity index of 0.197 indicated by DLS analysis by relatively narrow particle size distribution (Figure 5A). The particle size distribution curve showed a single dominant peak, with the formation of a comparatively homogeneous nanocomposite. The diffusion coefficient was measured as 1.2 μm^2^/s while the HMNCs transmittance was 65.2%, confirming acceptable particle dispersion. The HMNCs showed a mean zeta potential of −3.7 mV, with a distribution peak at −4.0 mV (Figure 5B). The single predominant peak observed in zeta potential distribution indicated the relatively uniform surface characteristics of the HMNCs. The surface charge and colloidal stability in the HMNCs were evaluated by zeta potential analysis. The relatively low magnitude of the zeta potential, together with the larger hydrodynamic size observed by DLS compared with the FESEM-derived particle size, suggests a tendency toward particle aggregation in aqueous dispersion.

**FIGURE 5:**
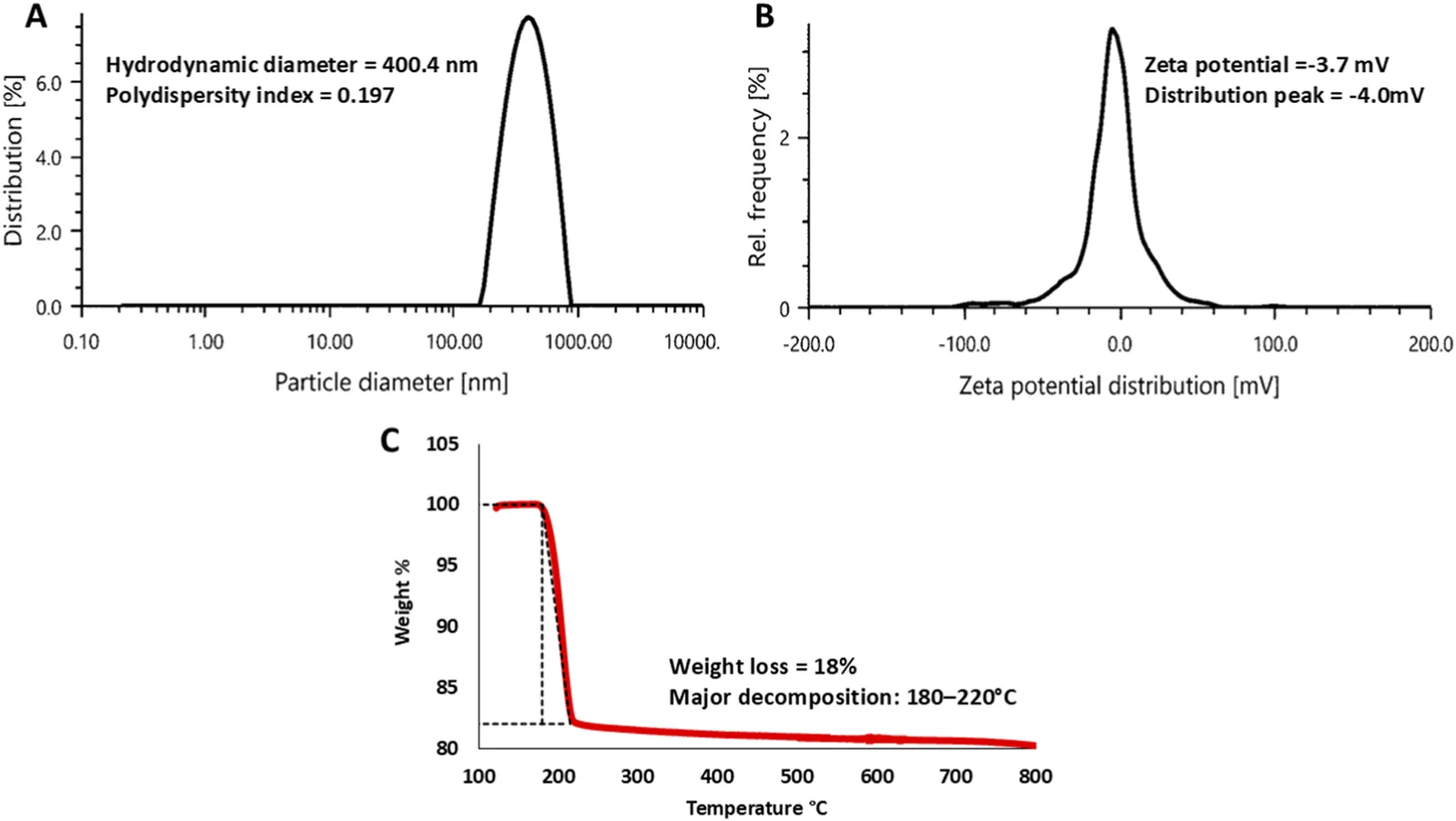
Physicochemical characterization of HMNCs: **(A)** DLS analysis showing the hydrodynamic particle size distribution of HMNCs, with a hydrodynamic diameter of 400.4 nm and a polydispersity index of 0.197; **(B)** Zeta-potential analysis showing the surface charge distribution of HMNCs, with a zeta potential of −3.7 mV; and **(C)** Thermogravimetric analysis (TGA) showing the thermal mass-loss profile of HMNCs, with an approximately 18% weight loss and major decomposition occurring between 180 °C and 220 °C.

Thermogravimetric analysis was performed to evaluate the thermal stability of the synthesized HMNCs. The thermogram of HMNCs showed weight loss between 180 °C and 220 °C (Figure 5C). The weight reduction was observed from approximately 100% of initial mass to 82% with a weight loss of 18%. It was observed that nearly 82% of the material remained stable up to 800 °C. The predominance of thermally stable inorganic mineral constituents within the nanocomposite plays a crucial role in retaining high residual mass even exposed to high temperatures.

The TGA profile demonstrated relatively limited mass loss over the temperature range investigated. In particular, only a small mass change was observed in the 180 °C–220 °C region, indicating limited thermal decomposition within this temperature interval. The overall residual mass at the end of the analysis represents the thermally stable inorganic fraction of the herbomineral nanocomposite. Therefore, the TGA results characterize the thermal mass-loss behavior and inorganic residue of the synthesized material, which is thermally stable and therefore safe for oral administration.

### 3.3 Isolation and screening of dental pathogens

A total of 61 bacterial isolates were recovered from the dental swab samples. Following purification and molecular identification, eight isolates (SE1–SE8) were identified as *S. epidermidis* and selected for subsequent investigations. The 16S rRNA gene sequences were deposited in the GenBank database under accession numbers PZ012429, PZ012432, PZ556812, PZ556813, PZ556814, PZ556815, PZ556816, PZ556817 respectively.

BLAST analysis of the 16S rRNA gene sequences showed 100% query coverage and 99.62% sequence identity with the selected *S. epidermidis* reference sequences, with an E-value of 0.0. The reference sequences used for comparison and phylogenetic analysis, together with their accession numbers and similarity values, are presented in Table 3. Maximum Likelihood phylogenetic analysis based on the Tamura–Nei model with 1,000 bootstrap replicates showed that isolates SE1–SE8 clustered with the selected *S. epidermidis* reference sequences (Figure 6). *Staphylococcus aureus* strain IraqYaAwSh22.6 (LC891134) was used as the outgroup. Bootstrap support values are indicated at the corresponding internal nodes of the phylogenetic tree.

**TABLE 3:** NCBI reference sequences used for 16S rRNA sequence comparison and phylogenetic analysis of *S. epidermidis* isolates.

S. No.	Reference strain	Accession no.	Query coverage (%)	Identity (%)	E-value	Role
1	*S. epidermidis* C4-3	OP852890.1	100	99.62	0.0	Reference
2	*S. epidermidis* T32-34	PV224742.1	100	99.62	0.0	Reference
3	*S. epidermidis* LaCa 250	CP190964.1	100	99.62	0.0	Reference
4	*S. epidermidis* HDRNA_03_K01	CP133677.1	100	99.62	0.0	Reference
5	*S. epidermidis* HDRNA_12_K01	CP133684.1	100	99.62	0.0	Reference
6	*S. epidermidis* TMDU-302	CP093188.1	100	99.62	0.0	Reference
7	*S. epidermidis* A4-4	OP852820.1	100	99.62	0.0	Reference
8	*S. epidermidis* Is01	CP130990.1	100	99.62	0.0	Reference
9	*S. epidermidis* UMCG388	CP064646.1	100	99.62	0.0	Reference
10	*S. aureus* IraqYaAwSh22.6	LC891134.1	100	99.62	0.0	Outgroup

**FIGURE 6:**
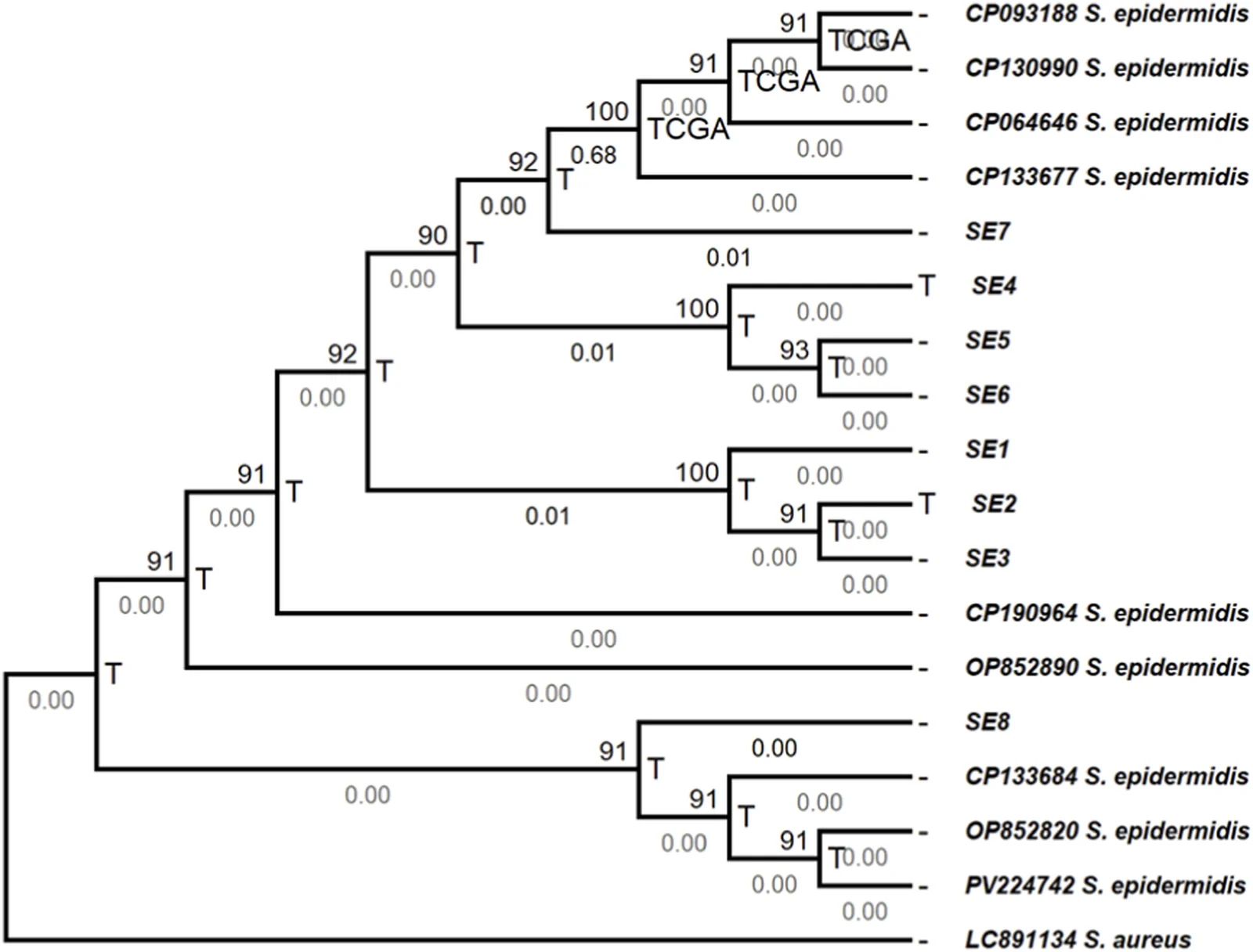
Phylogenetic analysis of *Staphylococcus epidermidis* isolates: Maximum-likelihood phylogenetic tree based on 16S rRNA gene sequences showing the evolutionary relationships of the eight isolates (SE1–SE8) with reference *S. epidermidis* strains. The tree includes *Staphylococcus aureus* (LC891134) as an outgroup. Numbers at the nodes indicate bootstrap support values, and the scale represents the estimated evolutionary distance.

The identified *S. epidermidis* isolates were subsequently evaluated for susceptibility against Norfloxacin, Enrofloxacin, Penicillin-G, Tetracycline, Gentamicin, Cefoperazone/Sulbactam, Ceftriaxone, and Ampicillin. Substantial variability in susceptibility patterns was observed among the isolates. All eight strains exhibited complete sensitivity to norfloxacin, indicating its high effectiveness against the tested oral isolates. Similarly, most isolates were sensitive to enrofloxacin, gentamicin, and cefoperazone/sulbactam, although isolated cases of intermediate susceptibility and resistance were detected.

In contrast, resistance to penicillin-G was observed in all eight isolates, suggesting widespread β-lactam resistance may exist among the isolated strains. Variable responses were also recorded for tetracycline, ceftriaxone, and ampicillin. Strain 4 exhibited the highest level of resistance, showing resistance to tetracycline, gentamicin, cefoperazone/sulbactam, and ceftriaxone, together with intermediate susceptibility to enrofloxacin. Strains 3, 5, and 8 also demonstrated resistance to multiple antibiotics, particularly β-lactam agents. Intermediate susceptibility to ceftriaxone was observed in strains 2, 5, and 7, while strains 1 and 7 displayed intermediate responses to tetracycline. Overall, the antibiotic susceptibility analysis demonstrated variable resistance patterns among the tested *S. epidermidis* isolates, with strain 4 exhibiting the highest level of resistance to penicillin-G, tetracycline, gentamicin, cefoperazone/sulbactam, and ceftriaxone, together with intermediate susceptibility to enrofloxacin. The universal resistance to penicillin-G and the occurrence of resistance to other clinically relevant antibiotics highlight the adaptive capacity of these organisms and their potential role as opportunistic pathogens in oral infections. These findings emphasize the need for alternative antimicrobial strategies capable of overcoming conventional antibiotic resistance. Consequently, the synthesized herbomineral nanocomposites (HMNCs) were further investigated as a potential antimicrobial agent against the isolated multidrug-resistant *S. epidermidis* isolates. The complete inhibition-zone measurements obtained for the eight isolates are presented in Table 4.

**TABLE 4:** Inhibition-zone diameters of the tested antimicrobial agents against *S. epidermidis* isolates.

Antibiotic	Disk potency	SE1	SE2	SE3	SE4	SE5	SE6	SE7	SE8
Values represent inhibition-zone diameters in mm									
Norfloxacin	10 µg	22	28	20	18	26	20	20	28
Enrofloxacin	5 µg	30	24	24	20	30	27	24	34
Penicillin-G	10 U	20	10	0	0	0	40	10	0
Tetracycline	30 µg	15	22	22	0	22	22	16	10
Gentamicin	10 µg	28	24	22	0	28	24	24	30
Cefoperazone/sulbactam	75/30 µg	30	22	18	15	27	29	22	28
Ceftriaxone	30 µg	34	16	0	0	18	0	16	24
Ampicillin	10 µg	40	27	10	24	10	24	24	18

### 3.4 Antibacterial activity of HMNCs in isolated *Staphylococcus epidermidis*

The antibacterial activity of the fabricated herbomineral nanocomposites (HMNCs) was evaluated in eight oral isolates of *S. epidermidis* (SE1–SE8) employing minimum inhibitory concentration (MIC) and minimum bactericidal concentration (MBC) assays. The MIC values of HMNCs were between 0.325 and 1.5 μg/mL, while MBC vales were observed as 0.325–3.125 μg/mL. Based on the MIC values of 8 isolates, SE4 and SE6 have shown the lowest MIC values of 0.325 μg/mL, indicating their high susceptibility to the HMNCs. Whereas the highest MIC value 0.7 μg/mL for SE1, 2, 3. The lowest MBC value was observed for SE6 (0.325 μg/mL), suggesting complete destruction of cells. Whereas the highest MBC value 0.7 μg/mL for SE5, 8.

The MBC/MIC ratios ranged from 1.00 to 2.15 across the tested isolates (Table 5), remaining below the commonly used threshold of 4 for distinguishing bactericidal from bacteriostatic activity. The HMNCs demonstrated antibacterial efficacy in all *S. epidermidis* isolates, with effective bactericidal property even at relatively low concentrations under the experimental conditions used in this study. The antibacterial activity observed in the present study may be associated with the physicochemical characteristics of the complete HMNCs formulation. The incorporation of silver within the mineral matrix was confirmed by the elemental characterization; however, the relative contribution of silver and the mineral components to the observed antibacterial activity was not separately determined.

**TABLE 5:** MIC, MBC and Tolerance level of HMNCs in different strains of *S. epidermidis*.

Bacterial strains	MIC (µg/mL)	MBC (µg/mL)	MBC/MIC ratio	Interpretation
SE1	0.7	1.5	2.14	Bactericidal
SE2	0.7	1.5	2.14	Bactericidal
SE3	0.7	1.5	2.14	Bactericidal
SE4	0.325	0.7	2.15	Bactericidal
SE5	1.5	3.125	2.08	Bactericidal
SE6	0.325	0.325	1	Bactericidal
SE7	1.5	1.5	1	Bactericidal
SE8	1.5	3.125	2.08	Bactericidal

The effect of HMNCs on the metabolic activity of *S. epidermidis* isolates obtained from dental caries-associated samples (SE1–SE8) was evaluated using a resazurin-based assay and compared with the antibiotic control (ampicillin). The untreated control groups showed differences in fluorescence intensity among the eight *S. epidermidis* isolates, reflecting variations in their baseline metabolic activity. Measurable fluorescence was also observed in the ampicillin-treated groups, indicating the presence of residual metabolically active bacterial cells following antibiotic exposure (Figures 7A–H). In contrast, HMNCs treatment resulted in a concentration-dependent reduction in fluorescence intensity across all eight isolates. At lower HMNCs concentrations (0.325–1.5 μg/mL), a partial reduction in metabolic activity was observed, whereas increasing the HMNCs concentration from 3.125 to 50 μg/mL resulted in a progressive decrease in fluorescence intensity. The greatest reduction in fluorescence was observed at the higher concentrations (25–50 μg/mL). Although the magnitude of the response varied among the isolates, all strains showed reduced resazurin conversion following HMNCs treatment. Overall, these findings demonstrate that HMNCs exposure was associated with a concentration-dependent reduction in the metabolic activity of *S. epidermidis* isolates compared with the untreated controls, with a pronounced effect observed at higher HMNCs concentrations.

**FIGURE 7:**
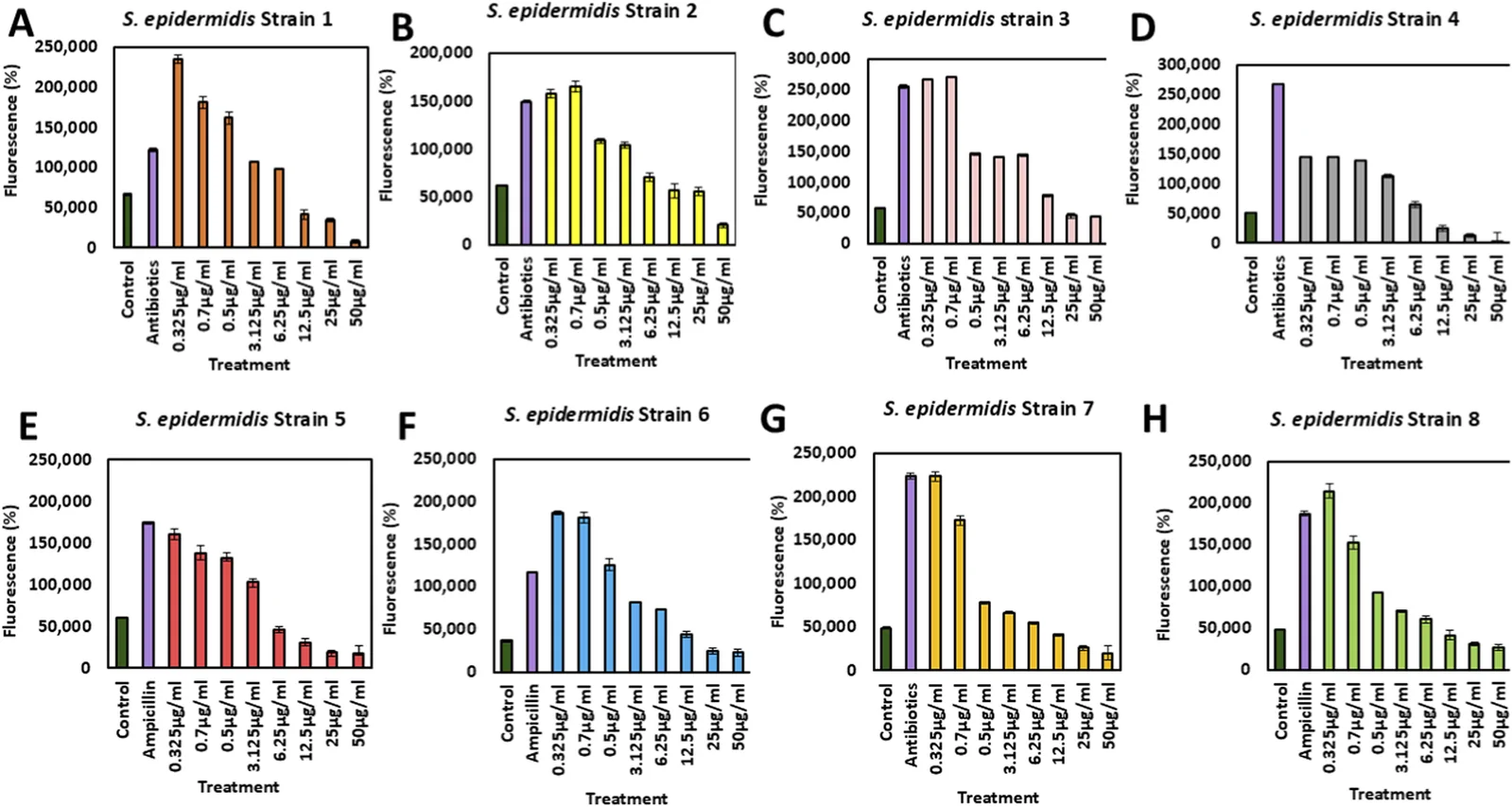
Resazurin assay showing the effect of HMNCs on the viability of *Staphylococcus epidermidis* isolates: **(A–H)** SE1–SE8 isolates compared with untreated control and antibiotic-treated groups. Data are presented as mean ± SD (n = 3). *p < 0.05, **p < 0.01, ***p < 0.001, ****p < 0.0001 compared with the untreated control.

The time-dependent antibacterial activity of HMNCs against eight *S. epidermidis* isolates (SE1–SE8) was evaluated by monitoring bacterial growth at 12, 24, 36, and 48 h. In all experiments, untreated controls maintained at 100% growth and compared with antibiotic and HMNCs treatment.

At 12 h, HMNCs treatment resulted in growth inhibition ranging from 55% to 78%, whereas the antibiotic-treated group exhibited only 11%–28% inhibition (Figure 8A). The percentage growth observed following antibiotic treatment was 79%, 83%, 76%, 76%, 89%, 72%, 82% and 81% respectively for SE 1- SE 8. Following HMNCs treatment, the corresponding growth values were 39%, 46%, 30%, 23%, 37%, 22%, 30% and 25%, respectively. The mean growth inhibition was approximately 69% for HMNCs-treated cultures compared with approximately 21% for antibiotic-treated cultures. These findings indicate greater inhibition of apparent bacterial growth following HMNCs treatment under experimental conditions.

**FIGURE 8:**
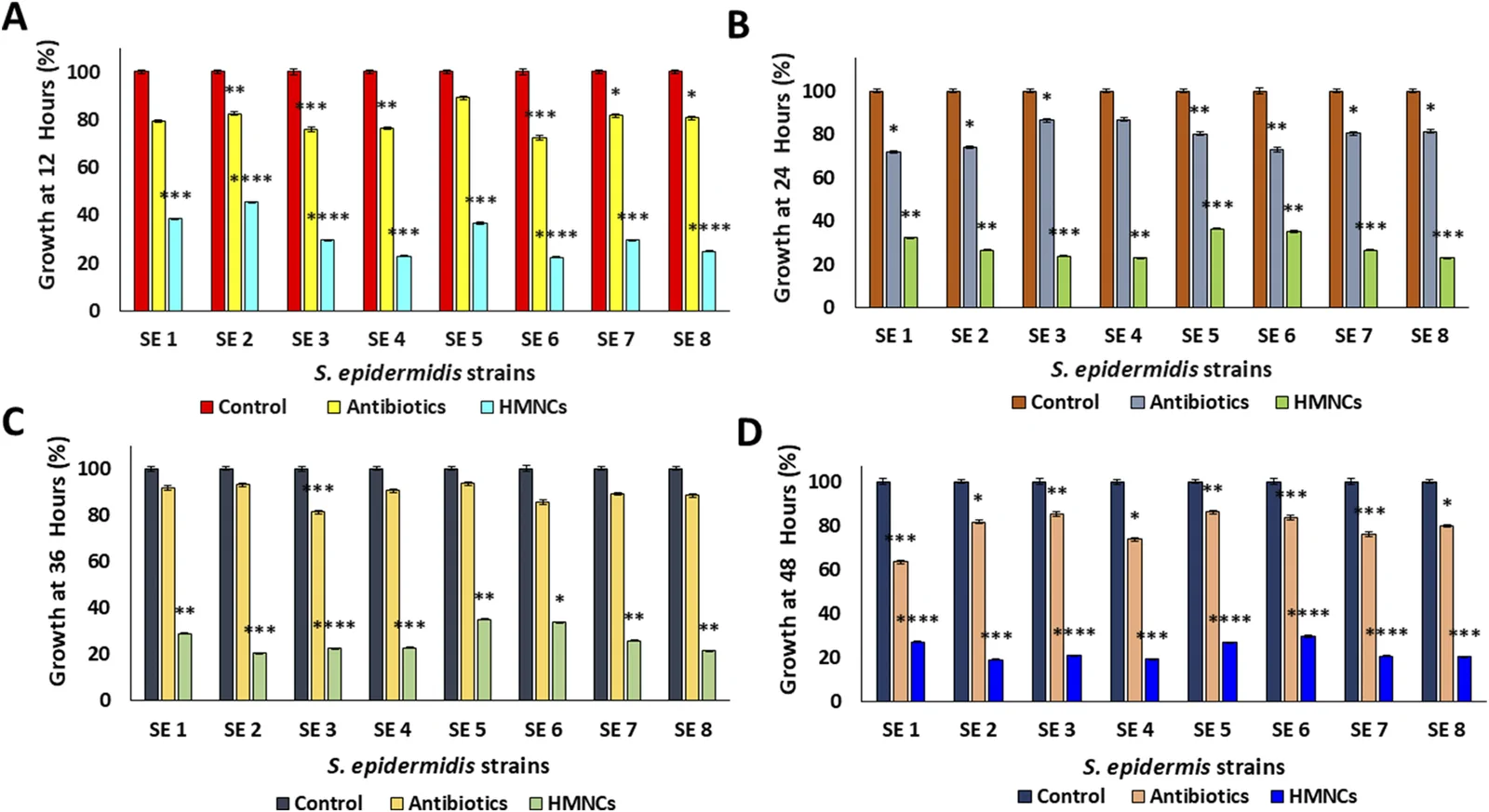
Antibacterial activity of HMNCs: Growth kinetics of *Staphylococcus epidermidis* isolates treated with HMNCs compared with the untreated control and antibiotic-treated groups. Bacterial growth was monitored at different incubation periods: **(A)** 12 h, **(B)** 24 h, **(C)** 36 h and **(D)** 48 h. Data are presented as mean ± SD (n = 3). Statistical significance was determined by comparison with the untreated control: *p < 0.05, **p < 0.01, ***p < 0.001, ****p < 0.0001 compared with the untreated control.

At 24 h, antibiotic treatment resulted in 13%–28% growth inhibition, whereas HMNCs treatment showed 64%–77% inhibition (Figure 8B). The percentage growth following antibiotic treatment was 72%, 74%, 86%, 87%, 80%, 73%, 80%, and 81% for SE1–SE8, respectively, whereas HMNCs-treated cultures exhibited 32%, 26%, 24%, 23%, 36%, 35%, 27% and 23% growth, respectively. The highest growth inhibition was observed for SE4 and SE8 (77%), followed by SE3 (76%) and SE2 (74%). Overall, HMNCs treatment produced a mean growth inhibition of approximately 71.8%, compared with 20.9% for antibiotic-treated cultures, indicating sustained inhibition of apparent bacterial growth at 24 h.

At 36 h, antibiotic-treated cultures showed 81%–94% residual growth, whereas HMNCs-treated cultures showed substantially lower apparent growth, ranging from 20% to 35% (Figure 8C). The percentage growth following antibiotic treatment was 92%, 93%, 81%, 91%, 94%, 85%, 89% and 88% for SE1–SE8, respectively, while HMNCs treatment resulted in growth values of 29%, 20%, 23%, 23%, 35%, 34%, 26% and 22%, respectively. The strongest growth inhibition was observed for SE2, SE3, SE4, and SE8. Overall, HMNCs treatment resulted in greater inhibition of apparent bacterial growth than the antibiotic treatment at 36 h.

At 48 h, HMNCs-treated cultures showed substantially reduced apparent growth, with growth values ranging from 19% to 30%, whereas antibiotic-treated cultures showed growth values ranging from 63% to 86% (Figure 8D). The percentage growth following antibiotic treatment was 63%, 82%, 85%, 74%, 86%, 83%, 76%, and 80% for SE1–SE8, respectively. Following HMNCs treatment, the corresponding growth values were 27%, 19%, 21%, 19%, 27%, 30%, 21% and 20%, respectively. SE2 and SE4 showed the lowest residual growth following HMNCs treatment. The mean growth inhibition was approximately 77.0% for HMNCs-treated cultures compared with 21.4% for antibiotic-treated cultures.

These findings indicate sustained inhibition of apparent bacterial growth by HMNCs over the 48-h observation period. Overall, HMNCs treatment consistently resulted in lower apparent bacterial growth than the antibiotic-treated cultures throughout the 48-h observation period. HMNCs treatment produced approximately 69.0%, 71.7%, 73.5% and 77.0% growth inhibition at 12, 24, 36, and 48 h, respectively, whereas the corresponding growth inhibition values for the antibiotic-treated cultures were approximately 20.7%, 20.9%, 10.9% and 21.4%. These findings demonstrate sustained inhibition of apparent bacterial growth following HMNCs treatment under the experimental conditions. The growth-kinetics experiment demonstrated sustained inhibition of apparent bacterial growth over the 48 h observation period. However, because the measurements were based on OD600 rather than viable CFU enumeration, the results should be interpreted as growth inhibition and cannot by themselves establish bactericidal activity or bacterial killing.

### 3.5 Insights on mechanistic investigation of HMNCs in targeting bacterial cells

#### 3.5.1 Oxidative stress-associated responses following HMNCs treatment

To examine whether HMNCs exposure was associated with oxidative stress-related responses, DCFDA fluorescence, antioxidant-related parameters, and oxidative damage markers were evaluated in *S. epidermidis* isolates. The antioxidant status of bacterial cells was assessed by measuring superoxide dismutase (SOD), catalase (CAT), and reduced glutathione (GSH), while oxidative membrane damage was evaluated through malondialdehyde (MDA) production. The results showed changes in antioxidant-related parameters and oxidative-damage-related markers following HMNCs treatment compared with the antibiotic-treated and untreated control groups.

HMNCs-associated changes in oxidative activity were evaluated using the DCFDA fluorescence assay (Figure 9A). HMNCs-treated bacterial cultures exhibited higher DCFDA fluorescence than the untreated and antibiotic-treated groups. HMNCs-treated bacterial cells exhibited markedly higher fluorescence intensities than both untreated control and antibiotic-treated groups. The untreated control group exhibited baseline fluorescence intensities of 11,698, 14,675, 39,921, 56,465, 7,396, 44,457, 5,656 and 32,949 RFU for SE1–SE8, respectively. The fluorescence intensities observed in the antibiotic-treated isolates were 8,629, 3,629, 29,283, 46,215, 14,154, 49,350, 55,239 and 53,827 RFU for SE1–SE8, respectively. In comparison, HMNCs-treated cells exhibited substantially higher fluorescence intensities of 18,486, 40,516, 42,666, 98,985, 40,978, 69,400, 74,043 and 81,437 RFU, respectively. Among the tested isolates, SE4 displayed the highest fluorescence intensity following HMNCs treatment, followed by SE8 and SE7. Because DCFDA fluorescence is not specific for a particular reactive oxygen species and the present experiment did not include probe-interference, cell-free, or antioxidant-rescue controls, the increased fluorescence should be interpreted as an oxidative-response-associated signal rather than definitive evidence of intracellular ROS generation. HMNCs exposure was associated with increased DCFDA fluorescence and changes in oxidative-damage-related parameters. However, because DCFDA is a non-specific oxidative probe and appropriate probe-interference, cell-free, antioxidant-rescue and viable-biomass normalization controls were not included in the present study, these findings should not be interpreted as definitive evidence of intracellular ROS generation or as proof of a causal role of oxidative stress in HMNCs-mediated antibacterial activity.

**FIGURE 9:**
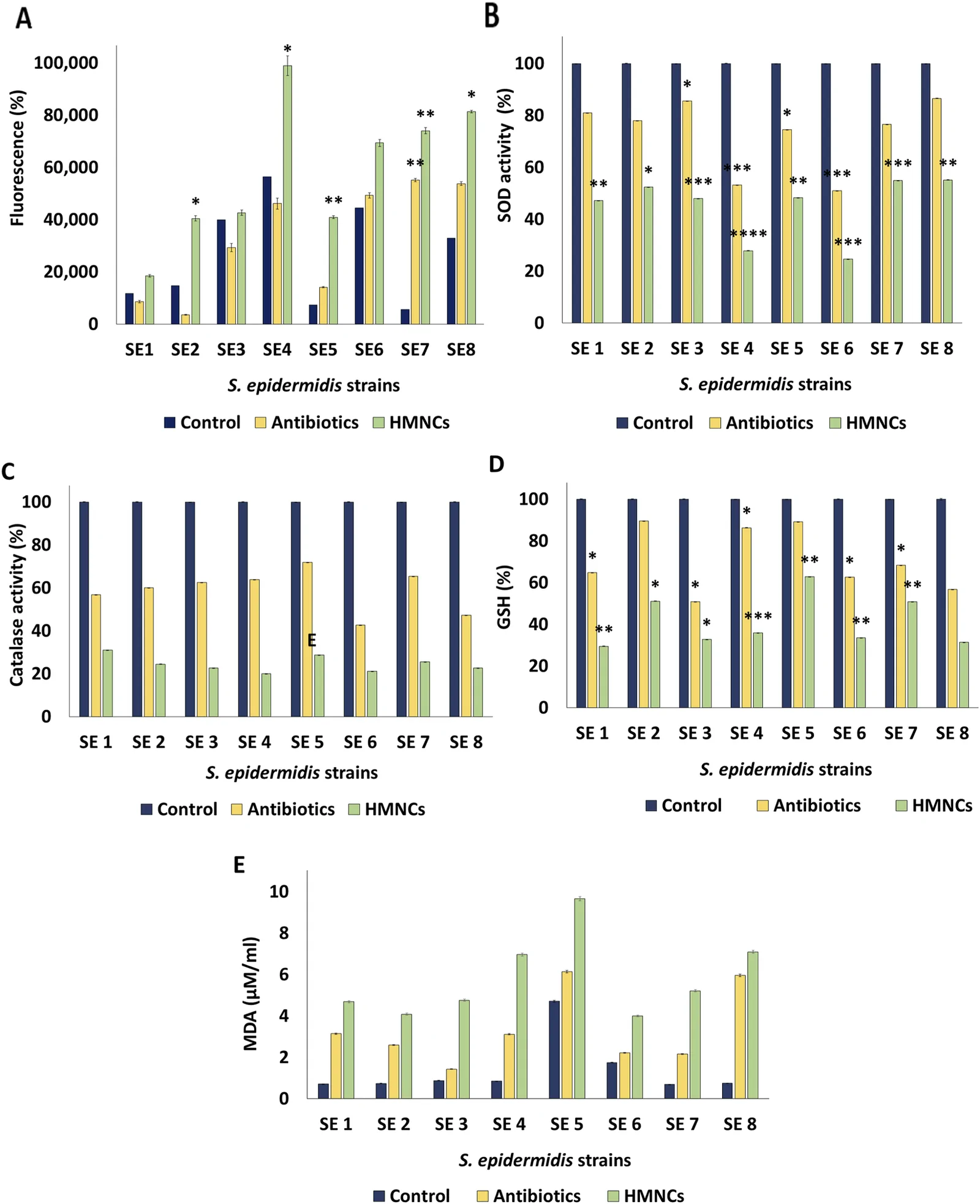
Oxidative stress and antioxidant response of *S. epidermidis* isolates following HMNCs treatment, compared with untreated control and antibiotic-treated groups: **(A)** Measurement of intracellular reactive oxygen species (ROS) by DCFDA assay; **(B)** Superoxide dismutase (SOD) activity; **(C)** Catalase (CAT) activity; **(D)** Reduced glutathione (GSH) levels; and **(E)** Malondialdehyde (MDA) levels. Data are presented as mean ± SD (n = 3). *p < 0.05, **p < 0.01, ***p < 0.001, ****p < 0.0001 compared with the untreated control.

Changes in SOD activity following HMNCs treatment were evaluated using the SOD assay (Figure 9B). The untreated control group exhibited the highest SOD activity across all tested isolates. In comparison, antibiotic-treated cells showed moderate reductions in SOD activity, with values of 81.0%, 77.9%, 85.5%, 53.2%, 74.4%, 51.0%, 76.6% and 86.6% for SE1–SE8, respectively. HMNCs treatment was associated with lower apparent SOD activity values compared with the antibiotic-treated and untreated groups, with relative values of 47.1%, 52.4%, 48.0%, 27.8%, 48.3%, 24.6%, 55.0% and 55.1% for SE1–SE8, respectively. The greatest reduction was observed in SE6 and SE4. HMNCs treatment was associated with reduced apparent SOD activity compared with the untreated and antibiotic-treated groups.

Changes in catalase (CAT) activity following HMNCs treatment were evaluated using the catalase assay (Figure 9C). The untreated control group maintained the highest catalase activity. Antibiotic-treated cells exhibited reduced catalase activity, with values of 56.8%, 60.1%, 62.5%, 63.8%, 71.8%, 42.7%, 65.4% and 47.3% for SE1–SE8, respectively. HMNCs-treated cultures showed lower apparent CAT activity values compared with the untreated and antibiotic-treated groups, with relative values of 31.0%, 24.4%, 22.5%, 19.9%, 28.8%, 21.1%, 25.5% and 22.6% for SE1–SE8, respectively. The greatest reduction in CAT activity was observed in SE4, followed by SE6 and SE3.

Relative reduced glutathione (GSH) levels were evaluated in *S. epidermidis* isolates following treatment (Figure 9D). The untreated control group maintained 100% GSH levels in all tested isolates. In contrast, antibiotic-treated cells exhibited GSH levels of 64.7%, 89.5%, 50.8%, 86.3%, 89.0%, 62.5%, 68.3% and 56.7% for SE1–SE8, respectively. HMNCs treatment was associated with lower relative GSH levels compared with the untreated control, with values decreasing to 29.5%, 51.1%, 32.7%, 35.8%, 62.9%, 33.5%, 50.9% and 31.3% for SE1–SE8, respectively. The greatest reductions in GSH content were observed in SE1, SE3, SE6, and SE8, whereas SE5 retained comparatively higher GSH levels among the tested isolates.

Changes in malondialdehyde (MDA), a marker associated with lipid peroxidation and membrane oxidative damage, were evaluated in *S. epidermidis* isolates following treatment (Figure 9E). HMNCs-treated bacterial cells exhibited higher MDA levels compared with both untreated and antibiotic-treated groups. The untreated control group exhibited MDA levels of 0.71, 0.74, 0.88, 0.84, 4.71, 1.75, 0.69 and 0.75 μM/mL for SE1–SE8, respectively. The MDA concentrations in HMNCs-treated cultures were 4.68, 4.08, 4.75, 6.96, 9.66, 4.00, 5.21 and 7.09 μM/mL for SE1–SE8, respectively. Among the tested isolates, the highest MDA level was observed in SE5 (9.66 μM/mL), followed by SE8 (7.09 μM/mL) and SE4 (6.96 μM/mL). In contrast, antibiotic-treated cultures exhibited intermediate MDA levels ranging from 1.43 to 6.13 μM/mL, while untreated control cultures exhibited substantially lower MDA levels ranging from 0.69 to 4.71 μM/mL.

Overall, HMNCs-treated *S. epidermidis* isolates exhibited increased DCFDA fluorescence, accompanied by changes in SOD, CAT and GSH levels and increased MDA production compared with the antibiotic-treated and untreated control groups. These findings indicate that HMNCs exposure was associated with oxidative-response and oxidative-damage-related changes in the tested bacterial isolates.

#### 3.5.2 Estimating the leakage of intracellular components into extracellular medium

The effect of HMNCs on bacterial membrane integrity was further evaluated by measuring the leakage of intracellular sugars, proteins and nucleic acids from *S. epidermidis* isolates.

On comparison with the untreated control and antibiotic-treated groups, HMNCs-treated bacterial cells exhibited markedly higher extracellular sugar concentrations in all tested isolates (Figure 10A). The extracellular sugar concentrations in the untreated control cultures were 15.7, 12.2, 10.3, 6.4, 2.9, 8.4, 5.7, and 3.8 μg/mL for SE1–SE8, respectively. Ampicillin treatment resulted in sugar leakage values of 17.6, 24.0, 15.0, 17.0, 8.7, 16.8, 7.3 and 6.9 μg/mL, respectively. In contrast, HMNCs treatment produced substantially higher sugar leakage values of 29.7, 39.0, 31.3, 21.3, 18.0, 24.6, 22.5 and 10.1 μg/mL for SE1–SE8, respectively. Among the tested isolates, the highest extracellular sugar leakage following HMNCs treatment was observed in SE2 (39.0 μg/mL), followed by SE3 (31.3 μg/mL) and SE1 (29.7 μg/mL), whereas SE8 exhibited the lowest leakage (10.1 μg/mL). Overall, HMNCs treatment consistently resulted in higher leakage of sugars than both the control and ampicillin-treated groups across all oral isolates.

**FIGURE 10:**
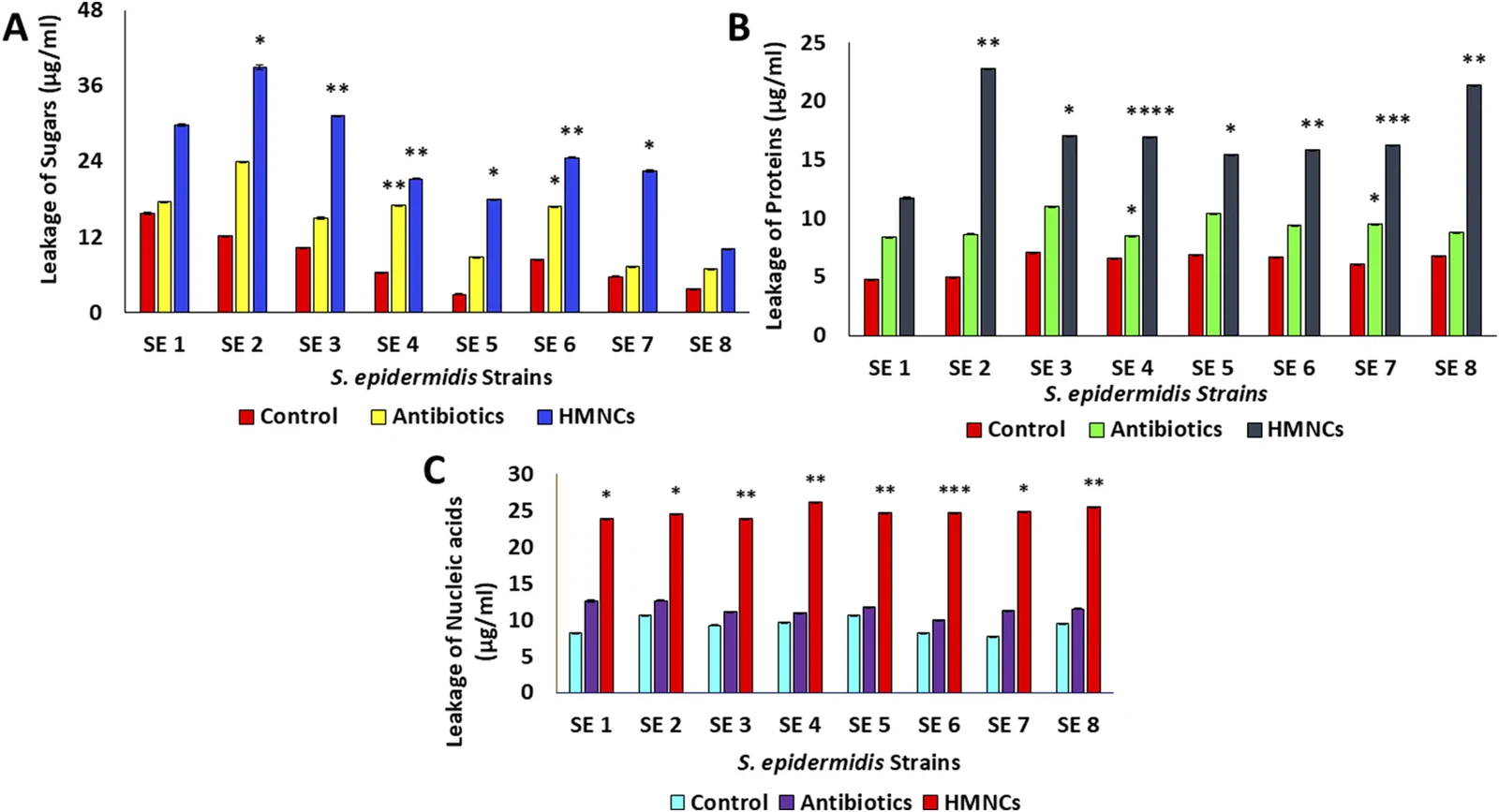
Assessment of membrane damage by measuring the leakage of intracellular components into the extracellular medium following HMNCs treatment compared with untreated control and antibiotic-treated groups: **(A)** Leakage of sugars; **(B)** Leakage of proteins; and **(C)** Leakage of nucleic acids. Data are presented as mean ± standard deviation (SD) from three independent experiments (n = 3). Data are presented as mean ± SD (n = 3). *p < 0.05, **p < 0.01, ***p < 0.001, ****p < 0.0001 compared with the untreated control.

The untreated control cultures showed protein leakage values of 4.7, 4.9, 7.0, 6.5, 6.9, 6.7, 6.1 and 6.8 μg/mL for SE1–SE8, respectively. Antibiotic-treated cultures exhibited protein leakage values of 8.4, 8.6, 11.0, 8.4, 10.4, 9.4, 9.5 and 8.8 μg/mL, respectively. In contrast, HMNCs-treated cultures demonstrated markedly elevated protein leakage values of 11.7, 22.8, 17.0, 16.9, 15.4, 15.8, 16.2 and 21.3 μg/mL for SE1–SE8, respectively (Figure 10B). Among the tested isolates, the highest protein leakage following HMNCs treatment was observed in SE2 (22.8 μg/mL), followed by SE8 (21.3 μg/mL) and SE3 (17.0 μg/mL), whereas SE1 exhibited the lowest leakage (11.7 μg/mL). Overall, HMNCs treatment consistently induced greater protein leakage than both the control and antibiotic-treated groups across all oral isolates.

The untreated control cultures showed comparatively low nucleic acid leakage, whereas ampicillin treatment resulted in a moderate increase in extracellular nucleic acid concentration. In contrast, HMNCs treatment increased levels of nucleic acid leakage across all tested isolates (Figure 10C). The extracellular nucleic acid concentrations in HMNCs-treated cultures ranged from 14.3 to 16.3 μg/mL, with the highest leakage observed in SE4 (16.3 μg/mL), followed by SE8 (15.7 μg/mL), SE7 (15.1 μg/mL), and SE5 (15.0 μg/mL).

Overall, HMNCs-treated cells exhibited substantially greater leakage of sugars, proteins and nucleic acid than both the control and antibiotic-treated groups, indicating extensive release of intracellular constituents from the bacterial cells.

### 3.6 Antibiofilm activity of HMNCs

#### 3.6.1 Crystal violet staining assay to quantify the biofilms

##### 3.6.1.1 Biofilm prevention activity of HMNCs against *S. epidermidis* isolates

The ability of HMNCs to prevent biofilm formation in *S. epidermidis* isolates was evaluated using the crystal violet assay (Figure 11A). Untreated control cultures were considered as 100% biofilm formation in all tested isolates. Antibiotic treatment resulted in partial inhibition of biofilm development, with biofilm formation percentages of 85.98%, 92.90%, 64.66%, 87.31%, 75.80%, 82.24%, 77.25% and 61.67% for SE1–SE8, respectively. In contrast, HMNCs treatment markedly inhibited biofilm establishment in all isolates, reducing biofilm formation to 33.27%, 23.50%, 20.17%, 26.51%, 21.54%, 20.66%, 26.57% and 17.56% for SE1–SE8, respectively. The strongest inhibition was observed in SE8 with 17.56% biofilm formation, followed by SE3 (20.17%), SE6 (20.66%), and SE5 (21.54%). Comparatively higher residual biofilm formation was observed in SE1 (33.27%), although biofilm percentage was substantially lower than corresponding antibiotic-treated group. Overall, HMNCs-treatment significantly lowers the biofilm formation than both antibiotic-treated and untreated control groups. The results demonstrate the strong ability of HMNCs to prevent the initial attachment, establishment and development of *S. epidermidis* biofilms across all tested oral isolates.

**FIGURE 11:**
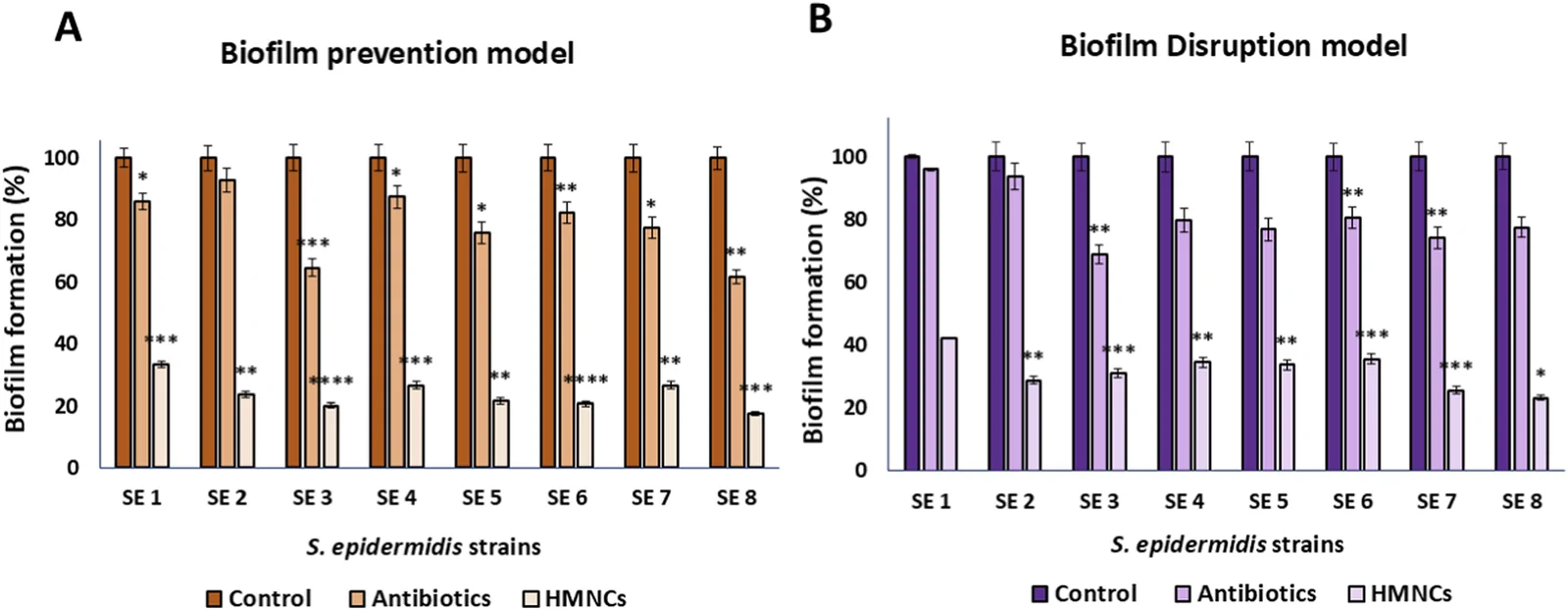
Biofilm prevention and disruption activity of HMNCs against S. epidermidis biofilms: **(A)** Biofilm prevention model, **(B)** Preformed biofilm disruption model. Data are presented as mean ± SD (n = 3). *p < 0.05, **p < 0.01, ***p < 0.001, ****p < 0.0001 compared with the untreated control.

##### 3.6.1.2 Biofilm disruption activity of HMNCs against preformed *S. epidermidis* biofilms

The ability of HMNCs to disrupt preformed biofilms of *S. epidermidis* isolates was evaluated using the crystal violet assay (Figure 11B). Untreated control cultures were considered as 100% biofilm biomass for all tested isolates. Antibiotic treatment resulted in partial biofilm disruption, with biofilm percentages of 87.85%, 93.63%, 69.06%, 79.70%, 76.79%, 80.48%, 74.11% and 77.45% for SE1–SE8, respectively. In contrast, HMNCs treatment markedly reduced the biomass of established biofilms in all tested isolates. The residual biofilm percentages following HMNCs treatment were 38.83%, 28.84%, 30.90%, 34.57%, 33.67%, 35.47%, 25.48% and 23.25% for SE1–SE8, respectively. The highest biofilm disruption was observed in SE8 (23.25%) and SE7 (25.48%), followed by SE2 (28.84%) and SE3 (30.90%). Although SE1 retained the highest residual biofilm biomass following HMNCs treatment (38.83%), biofilm formation remained substantially lower than that observed in the corresponding antibiotic-treated culture. These findings demonstrate the strong ability of HMNCs to disrupt established *S. epidermidis* biofilms across all tested oral isolates.

HMNCs exhibited antibiofilm activity in both the prevention and disruption models. However, the biofilm prevention model showed slightly higher efficacy, with residual biofilm formation ranging from 17.56% to 33.27%, compared with 23.25%–38.83% in the preformed biofilm disruption model. These findings indicate that HMNCs are effective at inhibiting the initial establishment of *S. epidermidis* biofilms than disrupting preformed biofilms, although substantial biofilm reduction was achieved in both models.

#### 3.6.2 FESEM analysis of biofilm morphology

FESEM analysis was performed to visualize the effect of HMNCs on the architecture of *S. epidermidis* strain 4 biofilms in both prevention and disruption models (Figure 12A). The untreated control exhibited dense bacterial colonization with a dense and multilayered biofilm structure occupying most of the coverslip surface. Numerous coccoid-shaped bacterial cells were observed in closely packed aggregates, indicating successful biofilm establishment and maturation.

**FIGURE 12:**
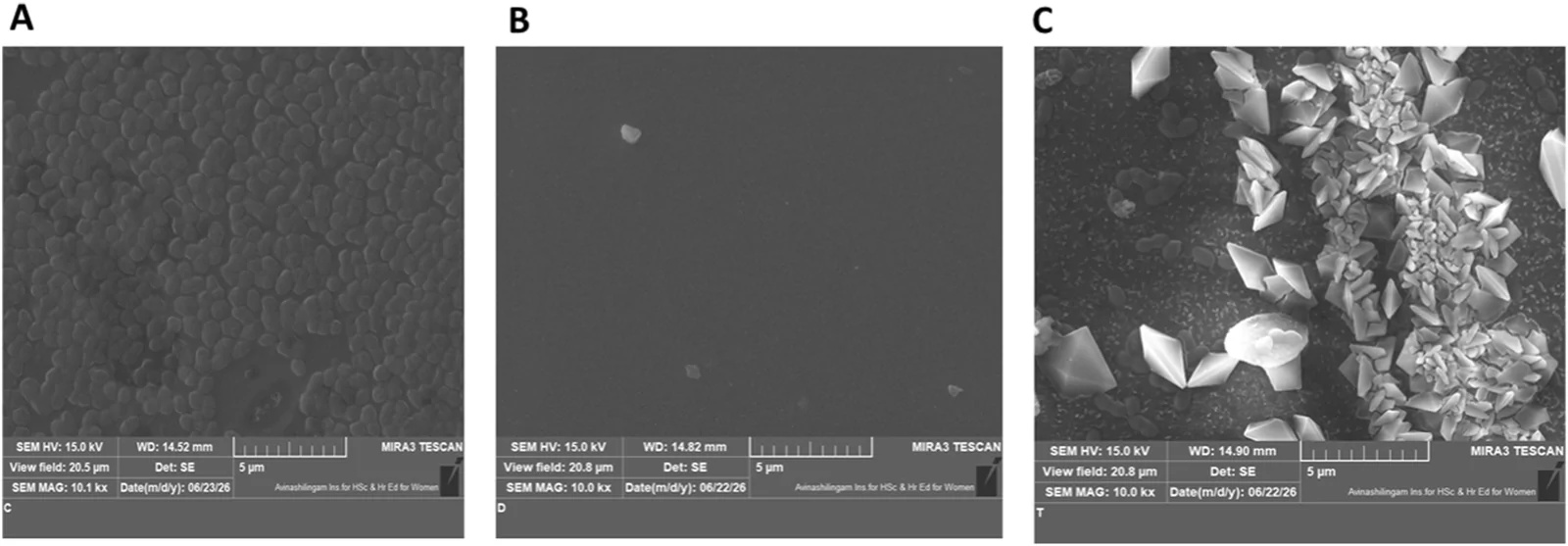
FESEM analysis of *S. epidermidis* biofilms comparing with HMNCs treatment in prevention and disruption model: **(A)** Untreated control showing dense biofilm formation and extensive bacterial colonization; **(B)** Biofilm prevention model showing marked inhibition of biofilm formation and reduced bacterial adherence following HMNCs treatment; **(C)** Preformed biofilm disruption model showing disruption of preformed biofilms with reduced biofilm density and bacterial aggregation.

In the biofilm prevention model (Figure 12B), HMNCs treatment significantly inhibited bacterial adhesion and biofilm formation. The treated surface appeared largely free from bacterial colonization, with only a few scattered deposits of debris on the coverslip.

In the preformed biofilm disruption model (Figure 12C), HMNCs treatment significantly disrupted preformed biofilms. Compared with the untreated control biofilms, the biofilm architecture appeared destroyed, with distinctly reduced bacterial colonies and increased exposed surface area. Although residual bacterial cells were still observed, the overall biofilm density was substantially lower than that of the untreated control.

Overall, FESEM observations demonstrated that HMNCs effectively inhibited biofilm formation and disrupted established biofilms. The prevention model exhibited greater reduction in bacterial colonization and biofilm coverage than the disruption model, indicating antibiofilm effect on preventing biofilm establishment than disrupting mature biofilms.

#### 3.6.3 Confocal laser scanning microscopy (CLSM) analysis of biofilms

Confocal laser scanning microscopy (CLSM) coupled with acridine orange/ethidium bromide (AO/EtBr) staining was employed as a complementary fluorescence-based approach to evaluate the effects of herbomineral nanocomposites (HMNCs) against *S. epidermidis* biofilms. The untreated control exhibited a dense biofilm, comprising 71.49% ± 5.09% AO-positive (predominantly viable) cells and 28.51% ± 5.09% EtBr-positive (membrane-compromised) cells, with a mean biofilm-covered area of 9750.78 ± 1,062.35 µm^2^ (Figure 13A).

**FIGURE 13:**
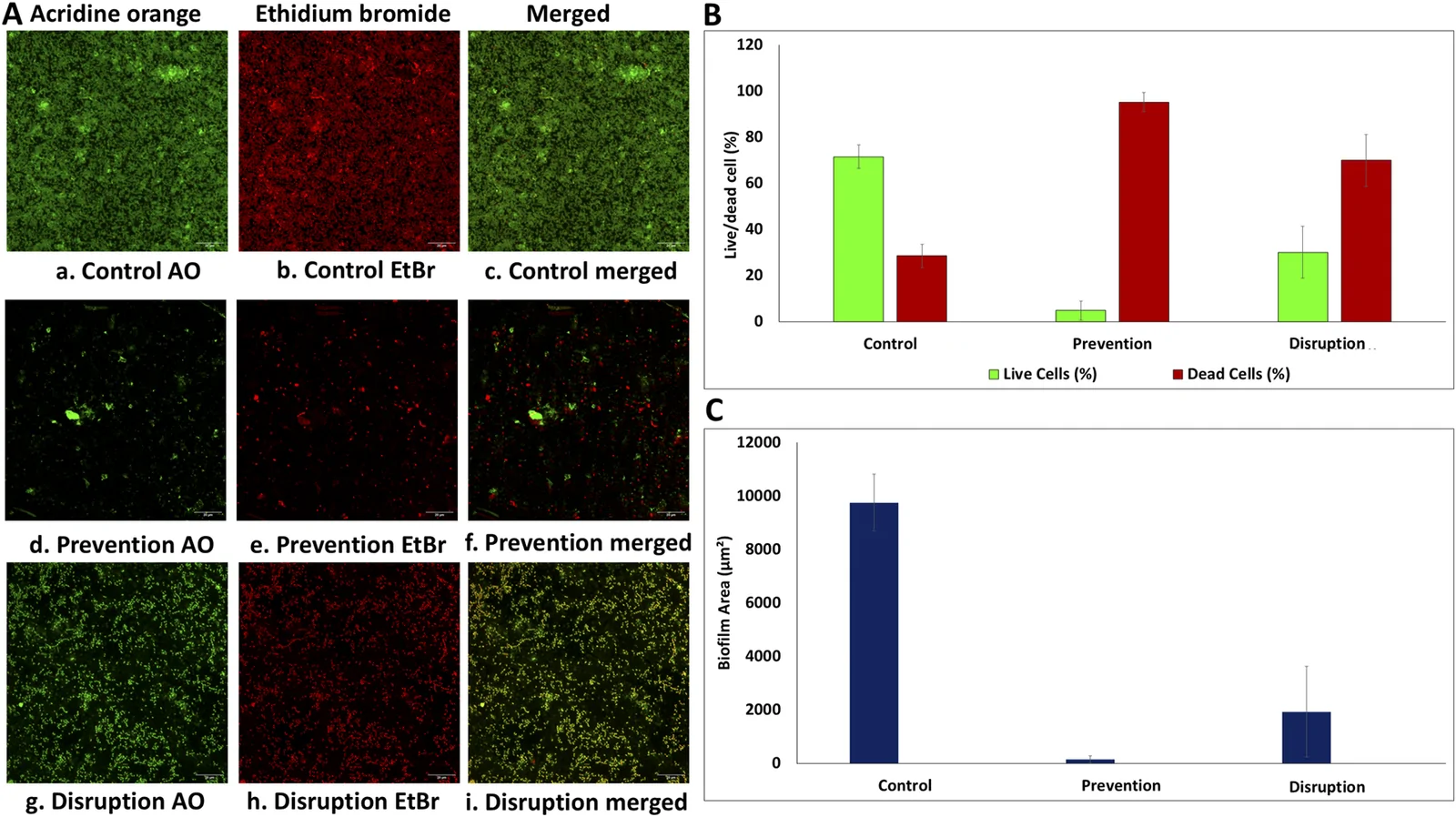
Confocal laser scanning microscopy (CLSM) analysis of *S. epidermidis* biofilms following HMNCs treatment in prevention and disruption model: **(A)** Representative AO/EtBr-stained CLSM images showing untreated control biofilms (A–C), biofilm prevention model (D–F), and preformed biofilm disruption model (G–I). Green fluorescence indicates viable cells (AO), while red fluorescence indicates dead or membrane-compromised cells (EtBr). **(B)** Quantitative analysis of live and dead cell populations. **(C)** Quantification of biofilm biomass expressed as biofilm-covered area (µm^2^). Data is presented as mean ± SD.

In the biofilm prevention model, HMNCs treatment reduced the proportion of AO-positive cells to 4.80% ± 4.14%, accompanied by an increase in EtBr-positive cells to 95.20% ± 4.14% (Figure 13B). Furthermore, the biofilm-covered area decreased to 144.07 ± 128.21 µm^2^, corresponding to a 98.52% reduction relative to the untreated control.

In the preformed biofilm disruption model, HMNCs treatment reduced the proportion of AO-positive cells to 30.07% ± 11.33% and increased the EtBr-positive fraction to 69.93% ± 11.33%. The residual biofilm-covered area was 1925.15 ± 1,698.73 µm^2^, corresponding to an 80.26% reduction relative to the untreated control (Figure 13C).

Overall, the CLSM observations indicated that HMNCs treatment reduced the measured biofilm-covered area and increased the proportion of EtBr-positive, membrane-compromised cells in *S. epidermidis* SE4 biofilms. The greater reduction in measured biofilm-covered area in the prevention model compared with the preformed biofilm disruption model suggests that HMNCs treatment was effective during biofilm establishment than against an already established biofilm. These CLSM findings provide complementary fluorescence-based evidence of changes in biofilm-associated viability and spatial coverage and should not be interpreted as a direct measurement of total biofilm biomass or complete biofilm disruption.

## 4 Discussion

The ICP-OES analysis confirmed that sulfur and calcium are the major elements in Kalnar Parpam. The mineral rich composition of KP provides insight into the inorganic framework. The presence of other trace amounts including iron, magnesium, sodium, potassium, and phosphorus indicates a composition of complex elements that are derived from the raw materials used for the preparation of Kalnar Parpam.

The FESEM analysis demonstrated that KP possesses a heterogeneous and structurally complex mineral matrix. The presence of elongated, plate-like and irregularly aggregated structures indicates that the material does not consist of morphologically uniform particles. Such morphological heterogeneity is expected for a traditional mineral-rich formulation containing multiple inorganic constituents with different physicochemical properties. The observed elongated and layered structures may be associated with the underlying silicate-rich mineral framework of Kalnar Parpam.

The XRD findings provide important structural evidence for the mineral nature of Kalnar Parpam. The strong reflection at 29.89° and the associated reflection at 36.47° are consistent with reflections reported for serpentine-type magnesium silicate. Previous crystallographic investigations of serpentine-related Mg–Si silicate have reported characteristic reflections at approximately 29.5° and 25.55°, supporting the assignment of these reflections to a Mg–Si silicate framework. The presence of additional reflections at 31.51°, 38.83°, 40.97°, 41.59°, 50.00°, 52.63° and 54.51° suggests that the sample is not adequately described by a single crystalline phase. Instead, these reflections may arise from overlapping contributions of different mineral phases and structurally related Mg–Si-containing phases. This interpretation is consistent with the minerals from Kalnar Parpam. Recently, the literature on serpentine minerals emphasizes that chrysotile, lizardite and antigorite can occur as structurally related Mg–Si phases and require XRD comparison with reference diffraction data for reliable differentiation (Şaşmaz et al., 2024).

The elemental composition of KP provides a mineral rich platform for the subsequent synthesis of Kalnar Parpam-based herbomineral nanocomposites (HMNCs). The presence of high levels of sulfur and calcium together with other trace minerals contributes towards its unique structural, functional, biological properties of the fabricated HMNCs. The mineral constituents identified in the HMNCs may influence surface properties, particle–cell interactions, or the local physicochemical environment. However, the present study did not separately evaluate the antibacterial contribution of individual mineral constituents. Previous studies have used metal nanoparticles as antibacterial agents, alone or in combination. For example, metal oxide nanoparticles, silver zinc oxide nanoparticles showed the efficacy against the *Pseudomonas aeruginosa* strain (Kamer et al., 2024).

The fabrication of HMNCs showed visible colour transformation from milky white to dark brown was the characteristic feature of silver containing nanostructures. The change in colour is one of the key optical properties of nanoparticles synthesis contributed by the reduction of silver ions. However, visual observation alone was insufficient to confirm the synthesis of HMNCs. Therefore, detailed physicochemical characterization by using FTIR, FESEM-EDX, HRTEM-SAED, XRD etc was subsequently accomplished to verify the morphology, nanostructure, and crystallinity of the synthesized HMNCs.

UV–Visible spectroscopic analysis provided the strong evidence for successful synthesis of silver based HMNCs by showing strong peak at 400 nm. Whereas the absorption peak of extract at 250 nm was due to the minerals and organic constituents that were present in herbomineral extract. The absorption peak around 400 nm was characteristic of the surface plasmon resonance (SPR) phenomenon associated with silver nanoparticles. The formation of SPR peak upon reduction of silver ions indicates the silver containing nanoscale structure. The substantial shift from 250 nm to 400 nm, indicates the modification of optical properties during the synthesis and provides preliminary spectroscopic evidence. The shift in UV Vis peak is consistent with the visual colour transformation being further verified by using FESEM-EDX, HRTEM-SAED and other instrumental analyses. In previous studies, green synthesis of silver nanoparticles using *Adhatoda vasica* leaf extract, where UV–Visible analysis confirmed the formation of AgNPs through a characteristic SPR band around 400–450 nm (Chaudhari et al., 2023). Recent research has also demonstrated that biosynthesized silver nanoparticles displayed SPR peaks within the characteristic silver nanoparticle region, validating nanoparticles synthesis through optical spectroscopy (Ibrahim et al., 2024).

The FTIR analysis confirmed the predominantly inorganic and mineral nature of Kalnar Parpam. The broad O–H stretching band observed around 3,600–3,550 cm^-1^ suggests the presence of structural hydroxyl groups and physically adsorbed water associated with the mineral matrix. The weak band near 1,620 cm^-1^ further supports the presence of bound moisture commonly observed in mineral-based formulations. The strong absorption band at 1,100–1,030 cm^-1^ region corresponds to Si–O–Si and Si–O stretching vibrations, representative the silicate phases which constitute a major component of the formulation. This observation is consistent with the Kalnar Parpam a traditional mineral origin and indicates the presence of silica-containing crystalline phases. FTIR peak present at 670 cm^-1^ and 596 cm^-1^ were associated with Si–O bending and metal–oxygen vibrations, respectively, suggesting the occurrence of Ca and Mg based mineral phases. FTIR spectrum well correlated with the ICP-OES results, which clearly confirms the existence of calcium, magnesium, and silicon, and with the XRD analysis also showed crystalline mineral constituents with multiple phases. The predominant presence of silicate and metal–oxygen vibrations and the absence of considerable organic functional groups, confirms the establishment of a stable inorganic, mineral based framework in Kalnar Parpam.

The FTIR analysis confirmed the successful synthesis of HMNCs through the integration of mineral matrix of KP. The broad O–H stretching at 3,560–3,400 cm^-1^ region suggests the presence of hydroxyl-containing compounds that may have participated in the reduction and stabilization of silver-containing nanostructures during synthesis. The absorption band by 1,630 cm^-1^ supports the presence of phytochemical residues acting as capping agents on the nanoparticles surface. Similar to KP extract, the prominent peak 1,100–1,030 cm^-1^ corresponds to Si–O–Si and Si–O stretching vibrations, demonstrating that the silicate-rich framework of KP was retained after nanocomposite formation. Similarly, the bands around 670 cm^-1^ and 590 cm^-1^ are characteristic of Si–O bending and metal–oxygen vibrations arising from calcium-, magnesium-, and silver-associated mineral phases. The retention of silicate-related bands together with the appearance of hydroxyl-associated functional groups indicates successful surface functionalization of the mineral matrix by plant-derived biomolecules. These findings correlate well with ICP-OES and EDX analyses, which confirmed the presence of Ca, Mg, Si, S, Fe, and Ag, and with XRD results demonstrating the crystalline mineral nature of the nanocomposite. Therefore, the FTIR results provide evidence that the synthesized HMNCs possess a stable silicate-rich mineral framework decorated with phytochemical functional groups that contribute to nanoparticle stabilization and functionality. In previous reported, Si–O and metal–oxygen vibrational bands in biosynthesized silver nanocomposites by *Flemingia wightiana* leaf extract were observed, confirming the retention of mineral structures after nanoparticle formation (Reddy et al., 2020). Similarly, in recent research, characteristic hydroxyl, silicate, and metal–oxygen functional groups in green-synthesized silver nanomaterials were suggested to contribute to nanoparticles capping and stability (Chauhan et al., 2026).

The FESEM images further confirmed the successful synthesis of nanoscale particles in the fabricated HMNCs. Based on the measurement, the particle size of HMNCs ranges from 43.83 to 127.53 nm demonstrating the formation of nanostructures well within the nanoscale system. The variation in the particles size may be due to the heterogenous nature of KP and differences in nucleation and growth during the synthesis of silver nanoparticles. The observation of extensive agglomeration is a common characteristic feature of nanomaterials because of high surface energy and strong interparticle interaction. Morphology with rough surface and clustered arrangement of nanocomposites effectively increases the surface area. These features enable greater interaction efficacy between the nanoparticles in composite matrix and microbial cells thereby enhancing its antimicrobial potential. Interestingly the nanocomposites with elongated crystalline structures suggest the co-existence of minerals that are derived from KP extract. The micrograph observation strongly confirms the heterogenous nanoarchitecture comprised of minerals and metals. FESEM results were well consistent with SPR peak of UV Vis spectroscopic analysis by providing morphological evidence supporting successful synthesis of herbomineral nanocomposites.

The EDX analysis offered direct evidence for the elemental composition of the HMNCs and confirmed the coexistence of minerals and silver within the nanocomposite matrix. The predominance of oxygen, calcium, and sulfur reflects the mineral-rich nature of the nanocomposite and was consistent with the composition of the reducing agent KP. Based on the comparison of the ICP-OES and EDX results further explain the transformation of KP into HMNCs. ICP-OES analysis of the KP extract indicated the presence S and Ca as major elements and Mg, Fe, Na, K, P as trace elements. EDX analysis of HMNCs confirmed the retention of mineral elements of KP Ca, S, Mg and Fe, while additionally detecting silver and Si. These findings demonstrate that the mineral framework of KP was highly preserved during synthesis and simultaneously functionalized through incorporation of silver. This transition from a mineral-rich Siddha herbomineral formulation to a silver-integrated nanocomposite provides strong evidence for the successful fabrication of HMNCs. In recent studies, observations on mineral-supported silver nanocomposites by *Archachatina marginata*, where EDX analysis confirmed the coexistence of silver with naturally occurring mineral elements and confirmed enhanced physicochemical stability and biological activity (Atanda et al., 2024). In previous reports on silver nanocomposite studies, EDX spectra confirmed silver as the major antimicrobial component together with oxygen and mineral-associated elements originating from biological or inorganic matrices. In biosynthesized silver nanoparticles derived from *Ocimum sanctum*, EDX analysis revealed Ag as the dominant element along with carbon and oxygen initiating from phytochemical capping agents, indicating successful nanoparticle (Mishra et al., 2023).

The XRD analysis of HMNCs confirmed as a highly crystalline structure containing multiple crystalline phases originated from mineral constituents of KP. Diffraction pattern indicates the presence of many sharp reflections indicating the ordering and crystallinity of the structure. The dominant peak was observed at 29.36° with other reflections at 31.60°, 33.61°, 36.91°, 41.08°, 43.88°, 48.27°, and 50.55° implies that the nanocomposite contains mineral-derived crystalline constituents originating from KP. Though there are certain reflections that resembled close proximity to FCC of silver (JCPDS- 04–783), diffraction pattern was predominantly dominated by mineral-derived phases. Even though the presence of silver was confirmed by EDX despite not being the dominant crystalline phase detected by XRD. The elemental analysis of ICP-OES and EDX analyses indicated the presence of calcium, sulfur, magnesium, silicon, and iron therefore the predominant reflection at 29.36° may be associated with calcium and magnesium containing mineral phases of KP. Previous studies have reported that the characteristic (104) reflection of calcite (CaCO_3_) occurs at approximately 29.4° (JCPDS card no. 05-0586/86–2339). Therefore, the dominant peak was likely associated with mineral constituents preserved during the Siddha purification, calcination processes and fabrication of HMNCs. Crystallite size of HMNCs was approximately calculated as 98–100 nm confirms the nanocrystalline nature. This result correlate well with FESEM analysis depicting the particle sizes ranging from 43.83 to 127.53 nm. The slight variation may be due to aggregation and polycrystallinity of HMNCs. Furthermore, the XRD findings are very consistent with EDX analysis confirming the presence of calcium, sulfur, magnesium, silicon, iron and silver in the HMNCs. In recent reports on mineral-supported silver nanocomposites synthesized using clinoptilolite-rich rock, where silver was detected by EDX but contributed only weak FCC reflections in XRD due to its dispersion within the mineral matrix (Kiradzhiyska et al., 2024). Previous research observed that silver-related reflections were less intense than those of the supporting matrix despite confirmation of silver by elemental analysis (Mishra and Nayar, 2014). The demonstration of plate or flakelike-structures of HMNCs was mainly contributed by the mineral derived framework inherited from Kalnar Parpam. The appearance of layered morphology was consistent with the silicate rich mineral composition of the precursor material. The irregular/polymorphic nature of HMNCs was well correlated with the FESEM images.

The SAED pattern of the HMNCs exhibited well-defined concentric diffraction rings rather than a diffuse halo, indicating the presence of crystalline domains within the material. The appearance of multiple diffraction rings indicates the presence of several randomly oriented nanocrystalline domains which are characteristic of polycrystalline nanocomposites. The observations were consistent with XRD findings demonstrating the existence of multiple crystalline phases and HRTEM results by displaying plate or flakelike structure confirming the successful formation of crystalline Kalnar Parpam-based herbomineral nanocomposites.

The XPS results demonstrate that the material possesses an oxygen-rich Ca–Mg–Si surface matrix containing C, S and surface Ag, indicating a chemically heterogeneous inorganic surface. The high-resolution Ag 3d XPS spectrum showed a characteristic spin–orbit doublet, with Ag 3d_5_/_2_ and Ag 3d_3_/_2_ components located at approximately 368.3–368.5 and 374.3–374.5 eV, respectively. The separation between the two components was approximately 6.0 eV. After calibration using the adventitious carbon C 1 s signal at 284.8 eV, these binding energies were consistent with reported values for metallic Ag^0^. The XPS findings therefore support the presence of metallic silver at the sample surface. Similarly, recent (Yuan et al., 2026) research reported Ag 3d_5_/_2_ and Ag 3d_3_/_2_ peaks around 368 and 374 eV, respectively, with a ∼6 eV separation, and assigned these features to metallic Ag^0^.

Dynamic light scattering analysis revealed that the synthesized HMNCs possessed a mean hydrodynamic diameter of 400.4 nm. The HMNCs exhibited a PDI of 0.197, indicating a relatively narrow distribution of hydrodynamic sizes under the measurement conditions. However, the PDI value alone does not establish complete particle homogeneity or colloidal stability. Considering the relatively large hydrodynamic diameter of 400.4 nm and the low zeta potential of −3.7 mV, the DLS results may reflect particle association or aggregation in the aqueous dispersion. The relatively narrow size distribution observed under the measurement conditions may contribute to consistency in the measured dispersion characteristics; however, particle size and aggregation state can influence the interaction of HMNCs with biological surfaces and may consequently affect their antimicrobial activity.

Zeta potential is a physicochemical parameter that provides information about HMNCs surface charge and colloidal stability. The synthesized HMNCs exhibited a negative zeta potential value of −3.7 mV, indicating the presence of negatively charged surface groups associated with herbomineral nanocomposites. These surface groups can absorb into the nanocomposite surface during synthesis and act as natural stabilizing agents.

The particle size of the dried HMNCs observed by FESEM was approximately 70.15 nm, whereas the hydrodynamic diameter measured by DLS in liquid suspension was considerably higher (400.4 nm). This difference can be attributed to the different measurement principles and sample states used for these techniques. FESEM analysis was performed on the dried HMNCs powder and provides direct visualization of the particle morphology and dimensions in the dry state. In contrast, DLS was performed on an HMNCs suspension in a liquid medium and determines particle size from fluctuations in scattered light arising from the Brownian motion of dispersed particles. Therefore, the DLS hydrodynamic diameter represents the effective size of the scattering entities in suspension and can be influenced by particle aggregation or association as well as the surrounding hydration layer. The zeta potential of the HMNCs was −3.7 mV, indicating a relatively low surface charge and limited electrostatic repulsion between particles. Thus, the DLS and zeta-potential results suggest a tendency toward particle association and limited colloidal stability under the tested dispersion conditions. Nevertheless, the FESEM observations demonstrate nanoscale particle dimensions in the dried HMNC powder. These findings highlight that particle dimensions observed in the dry state by electron microscopy may differ substantially from the hydrodynamic size measured for associated or aggregated entities in liquid suspension.

TGA thermogram shows the weight loss observed between 180 °C and 220 °C may be due to the removal of adsorbed moisture and decomposition of residual organic constituents in HMNCs. The minimal loss even at higher temperatures, indicates the thermal stability of HMNCs which were inherited from the mineral rich Kalnar Parpam. Further the data indicates the incorporation of inorganic phase with the nanocomposite matrix. This finding indicates the thermal robustness of HMNCs which can be harnessed for the potential biomedical applications. Previous studies demonstrated that mineral-supported silver nanocomposites using *Moringa oleifera*, *Eugenia roxburghii DC*, *Eucalyptus camaldulensis*, and *Terminalia arjuna exhibited* improved thermal stability due to their inorganic framework (Volcanes Moreno et al., 2025). Green synthesized silver nanocomposites by plant extracts showing initial organic decomposition and high temperature stability due to inorganic phases (Abbas et al., 2024). Mineral-supported silver nanocomposites exhibiting high residual mass and enhanced thermal stability for biomedical applications (Burduşel et al., 2018). The isolation of eight *S. epidermidis* isolates from dental samples focuses on the common incidence of this opportunistic pathogen within the oral environment. Even though *S. epidermidis* is a commensal organism of the skin and mucosal surfaces, it has been increasingly identified as an important pathogen correlated with biofilm-mediated infections and antimicrobial resistance. The antibiotic susceptibility analysis revealed noticeable resistance patterns among the isolates. All the eight isolates were observed to have complete resistance to penicillin-G implied the prevalence of β-lactam resistance within the isolated dental isolates. This finding was consistent with previous reports that *S. epidermidis* commonly develops resistance mechanisms, including β-lactamase production and alterations in penicillin-binding proteins, which reduce the efficacy of conventional β-lactam antibiotics. In contrast, all isolates were susceptible to norfloxacin, suggesting that fluoroquinolone-based therapy may still be effective against 8 strains. High levels of susceptibility were observed for enrofloxacin, gentamicin, and cefoperazone/sulbactam, with occasional intermediate or resistant phenotypes. This variability reflects the genetic diversity of *S. epidermidis* populations obtained from dental caries-associated sample sand their competence to acquire resistance through horizontal gene transfer and selective antibiotic pressure. Strain 4 exhibited the strongest resistance profile, revealing resistance to multiple antibiotics, including tetracycline, gentamicin, cefoperazone/sulbactam, and ceftriaxone. Strains 3, 5, and 8 displayed resistances to more than one antimicrobial agent. The existence of these multidrug-resistant isolates is clinically considerable because treatment options become increasingly inadequate as resistance expands across multiple antibiotic classes. The incidence of intermediate susceptibility to tetracycline and ceftriaxone in several isolates further suggests the potential development of future resistance under continued antibiotic exposure. The observed resistance patterns emphasize the growing challenge imposed by antimicrobial-resistant *S. epidermidis* in clinical settings. Since biofilm-forming staphylococci often exhibit enhanced tolerance to antimicrobial agents, the emergence of multidrug-resistant isolates may contribute to persistent infections and treatment failure. These findings emphasize the necessity for alternative therapeutic approaches capable of overcoming conventional antibiotic resistance.

The demonstrated prevalence of antibiotic resistance among the isolates provided a strong rationale for researching the antibacterial potential of the synthesized herbomineral nanocomposites (HMNCs). The existence of multidrug-resistant strains offers a clinically relevant model for evaluating novel antimicrobial materials and supports the development of HMNCs-based strategies as potential alternatives or adjuncts to traditional antibiotic therapy.

Based on the evaluation of antibacterial activity, the low MIC and MBC values of HMNCs against all *S. epidermidis* isolates indicate strong antibacterial efficacy with potent bactericidal property. The MBC/MIC ratios ranged from 1.00 to 2.15, suggesting a bactericidal pattern based on the absence of visibly detectable growth at the corresponding MBC concentrations. However, quantitative viable-cell enumeration was not performed. Importantly, the antibacterial activity was observed against isolates exhibiting resistance to one or more conventional antibiotics, highlighting the ability of HMNCs to effectively control clinically relevant and potentially multidrug-resistant strains. The observed activity was likely due to the combined antimicrobial effects of silver nanodomains and the bioactive mineral matrix derived from Kalnar Parpam. Overall, the results demonstrate that the synthesized HMNCs possess bactericidal activity against *S. epidermidis* and highlight their potential as antimicrobial material for future biomedical applications. Recent study reported that silver-decorated nanoparticles effectively inhibited *S. epidermidis* biofilms through direct interaction with bacterial cells and disruption of membrane integrity (Takahashi and Moriguchi, 2024). Previous studies demonstrated the antibacterial efficacy of green-synthesized silver nanoparticles by pear, quince, and apple extract from the leaves against *E. amylovora* bacteria, with MIC values substantially higher than those observed in the present investigation. The lower MIC and MBC values obtained for HMNCs suggest enhanced antibacterial potency (Khalifa et al., 2025).

The resazurin assay provides understanding into the physiological status of bacterial cells with subsequent HMNCs exposure at varying concentrations. The reduction of resazurin is mainly dependent on the active cellular respiration and metabolic processes that are actively involved during the survival of bacteria. The gradual decrease in the fluorescence intensity can be well correlated with the interference of HMNCs with the essential metabolic process of cell survival. Biological method of fungus mediated synthesized silver nanoparticles were shown to significantly reduce resazurin conversion in *Staphylococcus* species by disrupting cellular respiration and ATP production (More et al., 2023). The disturbance in the metabolic process may be associated with the combinatorial action of silver and mineral matrix of HMNCs. Moreover, the physico chemical characters of HMNCs majorly regulates the interaction of nanoparticles with bacterial cell membrane, proteins, enzymes and nucleic acids. The interaction between HMNCs and bacterial cells may interfere with regular enzymatic functions that regulate critical metabolic pathways that are required for cellular homeostasis and survival. Disruption of these pathways can compromise energy production, respiration, systematic pathways for cellular repair and stress-response etc., thereby decreasing the capability of bacterial cells to withstand the antibacterial pressure inflicted by HMNCs. The ability of HMNCs to affect the metabolic activity of all tested isolates, including strains that are exhibiting resistance towards conventional antibiotics. These features suggest that multiple antibacterial mechanisms of herbomineral nanocomposite support in increasing its effectiveness against resistant bacterial populations.

The growth kinetics study on the effect of HMNCs on all tested *S. epidermidis* strains displayed sustainable antibacterial activity throughout 48 h however the antibiotic-treated culture showed evidence of higher bacterial growth, implying decreased long-term inhibitory efficacy when compared with HMNCs treatment. The prolonged inhibitory effect of HMNCs is particularly noteworthy because several isolates showed resistance to conventional antibiotics during susceptibility testing. The ability of HMNCs to maintain antibacterial activity throughout the study period suggests a sustained mode of action that effectively suppresses bacterial proliferation and may reduce the likelihood of bacterial regrowth or persistence.

The antibacterial potency of HMNCs was attributed to their unique physicochemical characteristics revealed by characterization studies. The effective antibacterial activity of HMNCs was contributed by the mechanistic action of mineral constituents in KP and the silver incorporated nanostructures. Based on the ICP-OES and EDX analysis of KP and HMNCs, the nanocomposite is a mineral-rich platform rich in calcium and sulfur with trace amounts of magnesium, iron, sodium, potassium, and phosphorus.

The antibacterial activity of the HMNCs may be primarily contributed by the incorporated silver nanodomains, which are recognized to disrupt bacterial membranes, oxidative-response-related changes and interfere with bacterial cellular functions. In addition to silver ions the inherited mineral-rich Kalnar Parpam also plays an important role in imparting antimicrobial action. Calcium and sulfur would have enhanced particle–cell interactions and contribute to the biological activity of the nanocomposite by means of adhering to cells, disrupting the balance of the cells and breaking down the protective walls. Magnesium and iron can influence surface reactivity and may involve in oxidative processes that influence the bacterial viability. Sodium, potassium, phosphrous have a dual role: one in maintaining the structural integrity and the physicochemical characteristics of HMNCS, other to create an unfavourable environment for the survival of bacteria. Sodium and potassium may contribute to disturbing the balance of the ionic environment. Phosphorus containing phases may enhance biological interactions. The mineral constituents identified in HMNCs, including calcium, magnesium, iron, sodium, potassium, sulfur and phosphorus, may contribute to the structural and physicochemical characteristics of the nanocomposite and may influence its biological interactions. Although these mineral elements are not considered the primary antibacterial components of the HMNCs, their presence within the mineral matrix may contribute to the overall physicochemical properties of the formulation. However, the individual contributions of these mineral components to the observed antibacterial activity were not independently evaluated in the present study; therefore, their specific antimicrobial roles and any potential interactions with the silver-containing fraction cannot be established from the current data.

The nanoscale dimensions observed in FESEM and HRTEM analyses provide a large surface area that facilitates interaction between the nanocomposite and bacterial cells. Silver-containing nanostructures are known to disrupt bacterial membranes, alter membrane permeability, associated with oxidative-response-related changes, and interfere with essential cellular processes, ultimately resulting in bacterial death.

Furthermore, XRD and SAED analyses demonstrated the crystalline and polycrystalline nature of the HMNCs, while FTIR confirmed the presence of silicate, hydroxyl, and metal–oxygen functional groups. These unique structural features may improve particle stability and contribute towards sustained antibacterial action. The high thermal stability HMNCs further supports the strength of the mineral-rich nanocomposite system and points out its advantageous features for its potent applications.

The key observation noted in the study was that the antibacterial effect of HMNCs remained stable from 12 to 48 h without any subsequent treatment. Though antibiotic treatment cannot control the growth over the period of study. This finding provides a noteworthy result on unique contributions made by HMNCs including sustained release, constant antibacterial efficacy, prevention the resurgence and replication over time with action of silver and mineral-based nanocomposite. The sustained antibacterial activity observed during the growth-kinetics study may reflect the physicochemical properties of the complete HMNCs formulation. However, the present study did not determine whether this activity was specifically attributable to silver, the mineral matrix, or an interaction between these components. Prolonged antibacterial effects are necessary for combating opportunistic pathogens including *S. epidermidis*. Because these opportunistic pathogens are capable of developing antibiotic-resistant, persistent biofilms and device/implant associated infections.

The MIC, operational MBC endpoint, resazurin-based metabolic assay, and growth-kinetics results collectively demonstrate substantial antibacterial activity of HMNCs against the tested *S. epidermidis* isolates. The amalgamation of nanocomposites morphology, crystalline arrangement, silver incorporation, and mineral-rich composition contribute to function as a antibacterial agent. The observed antibacterial activity may involve contributions from both the silver-containing nanostructures and the mineral matrix; however, their individual contributions and possible synergistic interaction were not experimentally determined in the present study.

Recent research by (Singaravel et al., 2026) demonstrated that *Sesamum indicum* leaf mucilage can serve as a natural reducing and stabilizing matrix for the fabrication of ZnO nanoparticles with concentration-dependent bactericidal activity against resistant *S. aureus*. The present study demonstrates that the mineral-rich Kalnar Parpam matrix can function as a biogenic component during HMNCs fabrication with silver, producing a nanocomposite with pronounced antibacterial activity against clinical *S. epidermidis* isolates. The low MIC range (0.325–1.5 μg/mL) and MBC/MIC ratios of 1.0–2.15 observed in the present study further support the bactericidal nature of the HMNCs. Together, these findings support the broader potential of naturally derived matrices for developing sustainable metal-based antimicrobial nanomaterials, while the present HMNCs system offers the additional feature of integrating silver with a traditional mineral-rich herbomineral.

The results of oxidative stress assays indicate the induction of intracellular reactive oxygen species (ROS) to exert the antibacterial activity in *S. epidermidis*. Increased DCFH-DA fluorescence was observed following HMNCs treatment, indicating an increased intracellular oxidative response. These reactive oxygen species such as superoxide anions, hydrogen peroxide, hydroxyl radicals are highly reactive substances capable of damaging the bacterial cellular components including lipids, proteins, and nucleic acids. The observed DCFDA fluorescence, changes in antioxidant-related parameters, and oxidative-damage-associated responses are consistent with an oxidative response following HMNCs exposure. However, the present study does not establish whether oxidative stress is a primary mechanism, a consequence of cellular damage, or one of several responses associated with HMNCs exposure.

The oxidative-response-associated assays showed increased DCFDA fluorescence following HMNCs treatment. This increased fluorescence, together with changes in antioxidant-related parameters and oxidative-damage-associated responses, is consistent with an oxidative response following HMNCs exposure. DCFDA fluorescence is not specific for a particular reactive oxygen species and may be influenced by factors such as probe interaction, cellular damage, and optical interference. Therefore, the present findings should not be interpreted as definitive evidence of intracellular ROS generation. The present study also does not establish whether oxidative stress is a primary mechanism, a consequence of cellular damage, or one of several responses associated with HMNCs exposure.

In order to protect the cells from ROS damage, bacterial system uses antioxidant defence enzyme Superoxide dismutase (SOD). SOD provides first line of defense mechanism by converting superoxide radicals into hydrogen peroxide. Catalase (CAT) is another important antioxidant enzyme that detoxifies hydrogen peroxide by converting it into water and oxygen. In the present study, HMNCs-treatment exhibited a considerable reduction in SOD and CAT activity on comparison with antibiotic treatment and untreated controls. Decrease in SOD and CAT activity indicates the reduction in the cells capacity to neutralize superoxide radicals and detoxify hydrogen peroxide. As a result, ROS accumulates excessively inside the cell further inducing oxidative stress.

In addition to enzymatic antioxidants, major non-enzymatic antioxidant responsible for maintaining intracellular redox balance is reduced glutathione (GSH). Here also HMNCs-treated isolates displayed significantly lower GSH levels than antibiotic-treated and untreated control groups. The reduction of intracellular GSH indicates increased usage of antioxidant reserves to reduce the elevated level of ROS production. Reduced GSH levels compromises the capability of bacterial cells to maintain redox homeostasis, making them more susceptible to oxidative damage.

HMNCs exposure was associated with reduced apparent SOD and CAT activity and lower relative GSH levels. These changes may reflect alterations in cellular redox status; however, because the measurements were not normalized to viable biomass or total protein and potential interference by the nanocomposite was not systematically evaluated, they cannot be interpreted as definitive evidence of antioxidant-enzyme suppression.

The consequences of excessive ROS accumulation and decreased antioxidant defenses were further reflected by the increased malondialdehyde (MDA) levels observed following HMNCs treatment. MDA is a well-established end product of lipid peroxidation and serves as a sensitive indicator of oxidative membrane damage. The elevated MDA levels suggest that ROS generated upon HMNCs exposure attack membrane phospholipids, leading to lipid peroxidation, membrane destabilization and loss of membrane integrity. Such oxidative deterioration of the bacterial envelope was consistent with the increased leakage of intracellular sugars, proteins and nucleic acids observed in HMNCs-treated cells.

Collectively, the DCFDA, SOD, CAT, GSH and MDA findings indicate that HMNCs exposure is associated with a pronounced oxidative stress response in *S. epidermidis*. HMNCs-treated cells exhibited increased DCFDA fluorescence and MDA levels together with reduced SOD, CAT and GSH levels, suggesting disruption of intracellular redox homeostasis and increased lipid peroxidation. These oxidative stress-associated changes were accompanied by increased leakage of intracellular sugars, proteins and nucleic acids indicating compromised membrane integrity. However, the present findings demonstrate an association between oxidative stress, membrane damage and antibacterial activity rather than establishing that ROS generation is directly responsible for bacterial killing. The contribution of ROS to HMNCs-mediated bactericidal activity should therefore be considered a potential mechanism that requires further validation using ROS-scavenger experiments and appropriate probe-interference controls.

The resulting oxidative damage to membrane lipids and cellular macromolecules may contributes to membrane dysfunction, leakage of intracellular constituents, and ultimately bacterial cell death, thereby explaining the bactericidal activity of the synthesized HMNCs. Previous research has shown that increased DCFDA fluorescence and MDA levels, together with reduced GSH, SOD, and CAT activities, indicate severe oxidative stress-induced *S. aureus* and *P. aeruginosa* bacterial damage. These findings are consistent with recent reports demonstrating that silver nanoparticles using quercetin exert antibacterial activity through ROS-mediated pathways (Godakhindi et al., 2025).

The mechanistic action of HMNCs was studied by analysing the leakage of intracellular sugars, proteins, and nucleic acids in *S. epidermidis* isolates. In all tested strains, HMNCs treatment resulted in markedly higher leakage of these intracellular constituents compared with both the untreated control and ampicillin-treated groups, demonstrating massive disruption of bacterial membrane integrity. Among the tested isolates, highest sugar leakage was observed in SE2 and SE3 and the highest leakage of proteins in SE2 and SE8, whereas SE4 and SE8 showed the highest nucleic acid leakage, signifying increased susceptibility of these strains to HMNCs-induced membrane damage. The degree in difference of leakage patterns among the tested strains suggest differences in their susceptibility to HMNCs-induced membrane disruption.

The leakage of sugars into the extracellular environment is an early indication of cell membrane permeabilization and loss of homeostasis. Similar to sugars, proteins, enzymes, nucleic acids are normally present in the cytoplasm of the intact cell; Increased extracellular levels of these measured constituents may be consistent with altered membrane permeability; however, the present assays do not independently establish the extent or mechanism of membrane disruption. Compared with ampicillin treatment, HMNCs treatment was associated with higher levels of measured leakage, suggesting greater membrane-associated leakage under the tested experimental conditions. Ampicillin primarily inhibits bacterial cell-wall synthesis, whereas the observed leakage following HMNCs exposure may be consistent with membrane-associated effects. However, the present leakage assays do not by themselves establish direct membrane disruption. The observed leakage may also be influenced by differences in cellular biomass, assay specificity, or possible interactions of HMNCs with the analytical reagents. Previous studies have reported that silver nanoparticles can interact with bacterial cell envelopes and increase membrane permeability (Hochvaldová et al., 2024). The increased MDA-associated signal observed following HMNCs exposure is consistent with oxidative-damage-related changes; however, because the TBA-based assay is not specific for MDA, these results should not be interpreted as definitive evidence of lipid peroxidation.

The oxidative-response-related changes and membrane-associated effects of HMNCs may be associated with their physicochemical characteristics, including their irregular morphology and surface features. The observed changes in DCFDA fluorescence and oxidative-damage-related parameters suggest that HMNCs exposure may be associated with oxidative responses; however, the present experiments do not establish ROS generation as a causal mechanism of antibacterial activity. The presence of silver nanodomains within the herbomineral matrix of Kalnar Parpam offers multimode of action. The oxidative-response-related and membrane-associated changes observed following HMNCs exposure may be related to the physicochemical characteristics of the nanocomposite. The irregular morphology, rough surfaces, flake-like structures and sharp edges observed by electron microscopy could facilitate interactions with the bacterial cell envelope. However, the present study did not directly demonstrate that these morphological features caused membrane damage or oxidative responses. Interaction of nano sized particles with sharp edges induces destabilization of membrane architecture, alterations in the permeability and release the essential intracellular metabolites in the extracellular medium. Membrane damage may disturb the transport of nutrients, production of energy, enzymatic activity and other metabolic processes required for bacterial survival and to overcome stress conditions. Silver ions and other metals (Mg, Fe, Ca, Na, K) present in HMNCs may bind with intracellular biomolecules and disturb the normal functioning of the cell. Mineral elements in HMNCs such as magnesium, iron, calcium, sulfur, phosphorus, sodium and potassium, may enhance cellular stress and induce ionic imbalance, together contributing to bacterial dysfunction and cell death. From this leakage assay it was prominent that HMNCs possess capacity to compromise membrane integrity.

These findings of oxidative stress and membrane leakage were in agreement with MIC, MBC, resazurin reduction, and growth kinetics assays, which collectively showed antibacterial activity of HMNCs against the tested antibiotic-resistant *S. epidermidis* isolates. HMNCs treatment resulted in concentration-dependent control of bacterial metabolic activity, low MIC and MBC values, and sustained inhibition of bacterial growth over 48 h. The operational MBC/MIC ratios ranged from 1.00 to 2.15 based on the subculture endpoint used in this study. These findings indicate strong antibacterial activity; however, they should not be interpreted as definitive quantitative evidence of bactericidal kinetics. The observed antibacterial activity was associated with changes in oxidative-response parameters, membrane-associated leakage, metabolic activity and biofilm biomass under the experimental conditions. Based on these findings, a proposed mechanism of antibacterial action is presented in Figure 14, in which HMNCs exposure is associated with an increased oxidative response, changes in antioxidant-related parameters, membrane disruption, leakage of intracellular components, and subsequent bacterial cell death.

**FIGURE 14:**
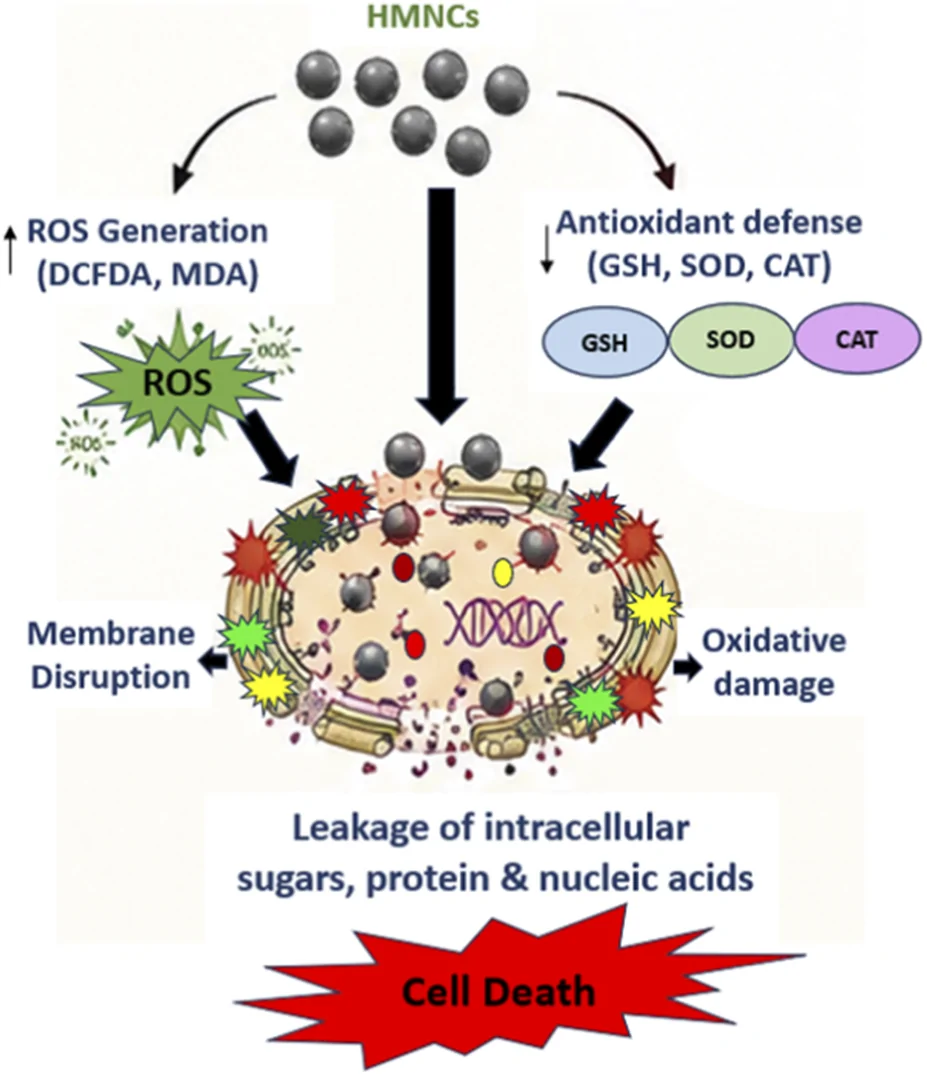
Proposed mechanism of the antibacterial action of HMNCs against *Staphylococcus epidermidis*. HMNCs exposure was associated with increased oxidative response, indicated by DCFDA fluorescence and MDA levels, together with changes in antioxidant-related parameters (GSH, SOD and CAT). These responses may contribute to membrane disruption, oxidative damage, and leakage of intracellular components, including sugars, proteins, and nucleic acids, ultimately contributing to bacterial cell death.

The crystal violet assay supported that HMNCs possess significant anti-biofilm activity against all tested *S. epidermidis* isolates in both biofilm prevention and preformed biofilm disruption models. HMNCs treatment resulted in a substantial reduction in biofilm compared with antibiotic-treated and untreated control groups, indicating reduction of biofilm biomass under both prevention and preformed-biofilm disruption conditions. Although HMNCs are effective in both models, slightly higher inhibition was achieved in the prevention model.

Biofilm formation is one of the major virulence features of *S. epidermidis* and plays an essential role in persistent, chronic and medical device-associated infections. The process of biofilm formation involves initial adhesion of bacterial cells to the surface, microcolony formation, maturation, and production of extracellular polymeric substances (EPS). EPS are well-known agents that provide structural stability, integrity, and protection against antimicrobial agents. Therefore, the prominent reduction in S. *epidermidis* biofilm under HMNCs treatment picturizes the impairment in biofilm formation as well as maintenance. The observed antibiofilm activity of HMNCs may be associated with interference with bacterial adhesion and/or extracellular polymeric substance (EPS) matrix development; however, these specific effects were not independently evaluated in the present study. Extracellular DNA is an important structural component of the EPS matrix and contributes to biofilm stability and bacterial adhesion. ROS-mediated damage and membrane disruption induced by HMNCs may reduce eDNA availability, thereby weakening biofilm architecture. In previous research, studies have highlighted that silver nanoparticles by metallic nature can destabilize EPS matrices through oxidative stress-mediated degradation of polysaccharides, proteins and extracellular DNA in *S. aureus* (Li et al., 2024).

The demolition of preformed biofilms by HMNCs treatment is particularly noteworthy because mature biofilms are naturally difficult to eradicate. Bacterial cells dwelling inside the biofilms are well protected by the EPS matrix, which limits the penetration of antibiotics and increases tolerance to antimicrobial agents. This phenomenon was clearly shown in this research work, where antibiotic-treated cultures retained considerably higher biofilms than HMNCs-treated groups in both prevention and disruption models. The activity of HMNCs suggests their competence to overcome the bacterial protective mechanisms associated with biofilm-mediated antimicrobial resistance.

The antibiofilm activity of HMNCs may also be associated with the antibacterial effects observed in mechanistic studies. Biofilm formation requires active bacterial metabolism, cellular communication and continuous production of extracellular matrix. HMNC exposure was associated with membrane-associated damage, oxidative-response changes, leakage of intracellular constituents, and suppression of bacterial metabolic activity. These effects may collectively impair bacterial physiological functions, thereby contributing to the inhibition of biofilm formation and disruption of preformed biofilms. The observed reduction in bacterial viability within the biofilm following HMNC treatment may further contribute to decreased biofilm biomass and stability.

Overall, the findings demonstrate that HMNCs inhibited biofilm formation and reduced preformed biofilm biomass in *S. epidermidis* isolates. The observed antibiofilm activity, together with the associated changes in bacterial metabolic activity, oxidative response, membrane-associated damage and leakage of intracellular constituents, suggests that these effects may collectively contribute to the prevention and disruption of biofilms. These findings highlight the potential of HMNCs as a Siddha-inspired antimicrobial material for further investigation as an antibiofilm strategy against persistent biofilm-associated oral infections. A recent study reported significant biofilm inhibition by a silver nanoformulation prepared using propolis extract against *P. aeruginosa*, supporting the potential contribution of silver-based nanomaterials to antibiofilm activity (Shiralizadeh et al., 2025). Similarly, silver-decorated nanoparticles have been reported to reduce *S. epidermidis* biofilms through interactions with biofilm-associated bacterial cells (Takahashi and Moriguchi, 2024). Collectively, these findings support further investigation of HMNCs as a potential antibiofilm material for oral healthcare applications.

The FESEM observations further confirmed the antibiofilm efficacy of HMNCs against *S. epidermidis* isolates by showing the clear difference between the control and both models. The control showed packed coccoid cell arranged as compact biofilm. The biofilm is the characteristic feature of Staphylococcal biofilm which act as protective barrier to protect from environmental stress factors. In biofilm prevention model, there was marked disruption and reduction of biofilm biomass. This was due to the action of HMNCs treatment, which completely prevents bacterial growth hence the biofilm was not formed. In the disruption model, HMNCs can effectively disrupt the preformed biofilm by showing a marked reduction in the biofilm adhesion. The morphological changes observed by FESEM in the untreated and HMNC-treated biofilms were in agreement with the crystal violet staining and CLSM findings, providing complementary evidence of HMNC-associated biofilm inhibition and disruption.

A previous study of the EPS rich biofilm observed in untreated *S. epidermidis* biofilm is consistent with the SEM observations, which reported bacterial cells interconnected by microvesicles and nanotube-like structures. HMNCs treated biofilms exhibited substantial disruption of bacterial aggregation and EPS organization, indicating effective prevention and disruption of biofilm architecture (Takahashi et al., 2021). CLSM analysis using AO/EtBr staining provided direct visual evidence of the antibiofilm activity of HMNCs against *S. epidermidis*. The untreated control biofilms exhibited dense biofilm structures with a predominance of live cells, indicating active and viable biofilm communities. In contrast, HMNCs-treated biofilms showed a marked reduction in live cells together with a substantial increase in dead cells, confirming the antibiofilm effect of the nanocomposites. Previous studies reported that biosynthesized silver nanoparticles from *Skimmia laureola* extract significantly decreased *S. aureus* viable biofilm-associated cells and disrupted biofilm architecture, as evidenced by CLSM imaging (More et al., 2023).

The prevention model demonstrated a greater reduction in viable cells and biofilm coverage than the disruption model, suggesting that HMNCs are effective at preventing biofilm establishment than eliminating mature biofilms. This observation is expected because mature biofilms possess a well-developed extracellular matrix that provides protection to embedded bacterial cells and reduces susceptibility to antimicrobial agents.

The recent study of (Singaravel and Murugan, 2025) reported that green-synthesized AgNPs prepared using *Musa* AAB stem juice exhibited significant antibiofilm activity against levofloxacin-resistant *S. aureus*, with biofilm reduction of up to 72.5% at a sub-MIC concentration. Similarly, the present HMNCs demonstrated substantial inhibition of biofilm formation and disruption of established *S. epidermidis* biofilms, supporting the potential of biologically fabricated silver-based nanomaterials as effective antibiofilm agents. However, the present HMNCs differ from the reported AgNPs by incorporating a multifunctional herbomineral platform rather than a conventional plant-mediated AgNP system.

Despite this protective barrier, HMNCs were able to significantly reduce the viability and coverage of preformed biofilms, indicating effective disruption of established biofilm communities. The effective activity of HMNCs compared with antibiotic treatment further highlights their potential for controlling biofilm-associated infections caused by *S. epidermidis*.

The CLSM findings are consistent with the crystal violet assay and resazurin metabolic activity assay, which also demonstrated significant inhibition of biofilm formation and reduction in biofilm viability following HMNCs treatment. Overall, the results confirm that HMNCs effectively suppress biofilm development and compromise the survival of biofilm-associated bacterial cells, supporting their potential application as antibiofilm agents against *S. epidermidis* infections.

The disruption of preformed biofilms by HMNCs treatment is particularly noteworthy because mature biofilms generally exhibit increased tolerance to antimicrobial treatment. In the present study, HMNCs treatment substantially reduced the residual biofilm biomass in the preformed-biofilm model, as demonstrated by crystal violet staining. CLSM analysis of SE4 further showed a reduction in biofilm-covered area together with an increased proportion of membrane-compromised cells following treatment. These findings indicate that HMNCs can disrupt established biofilm architecture and reduce associated biomass; however, they do not by themselves establish complete elimination of viable biofilm-associated bacteria.

Overall, the findings indicate that HMNCs effectively inhibit biofilm establishment and substantially reduce/disrupt preformed biofilm biomass in *S. epidermidis* isolates. The crystal violet assay demonstrated these effects across the tested isolates, while FESEM and CLSM provided complementary morphological and fluorescence-based evidence in SE4. Further studies incorporating biofilm-associated viable-cell enumeration and MBEC determination are warranted to establish the extent of viable biofilm elimination.

The difference between the crystal violet and CLSM findings should be interpreted in the context of their different analytical endpoints. Crystal violet staining estimates retained attached biofilm biomass, whereas CLSM analysis assesses fluorescence-defined biofilm-covered area and AO/EtBr-associated cellular signals in representative microscopic fields. Therefore, the percentage reduction obtained from CLSM should not be considered directly equivalent to the percentage reduction in crystal-violet-stained biomass. The observed difference may also reflect biofilm heterogeneity, field selection, and differences in the microscopic area analyzed.

### 4.1 Limitations

The characterization of HMNCs has some limitations. Although FESEM/HRTEM, EDX, XRD, XPS, DLS, zeta-potential and ICP-OES provided complementary information on the morphology, elemental composition, crystalline phases, surface chemistry, dispersion characteristics and silver content, these analyses do not definitively establish a structurally integrated nanocomposite. The larger DLS hydrodynamic diameter compared with the FESEM/HRTEM particle dimensions may also indicate particle aggregation or association in suspension.

A limitation of the antibiotic susceptibility testing was the absence of a standardized ATCC/MTCC quality-control reference strain. Future studies will incorporate appropriate reference strains to strengthen quality control and reproducibility of the susceptibility testing. Batch-to-batch reproducibility was also not independently assessed because the present study was performed using a single prepared batch of HMNCs. Future studies will evaluate independently prepared batches to establish reproducibility.

The interpretation of oxidative-response and antioxidant-related assays is limited by the non-specific nature of DCFDA fluorescence and the absence of dedicated probe-interference, antioxidant-rescue, and time-dependent controls. In addition, SOD, CAT, and GSH were expressed as relative values rather than normalized absolute activities. Therefore, these findings are interpreted as oxidative-response-associated changes following HMNCs exposure rather than definitive evidence of ROS-mediated antibacterial activity or direct suppression of antioxidant defenses.

The difference between the crystal violet and CLSM findings should be interpreted in the context of their different analytical endpoints. Crystal violet staining estimates retained attached biofilm biomass, whereas CLSM assesses fluorescence-defined biofilm-covered area and cellular signals in representative microscopic fields. Therefore, the percentage reduction obtained from CLSM should not be considered directly equivalent to the percentage reduction obtained from crystal-violet staining.

An important limitation is that component-specific controls were not included to determine the individual contribution of the silver and mineral fractions to the antibacterial activity of HMNCs. Silver-ion release and equivalent-silver normalization were also not evaluated. Therefore, the individual contributions of silver, mineral components and their possible interactions cannot be clearly distinguished. Future studies will incorporate appropriate AgNO_3_-only and KP-only controls, silver-release measurements, and equivalent-silver normalization.

Finally, comprehensive biocompatibility and toxicological evaluation was beyond the scope of the present study. Therefore, the findings should be considered preliminary evidence of antimicrobial and antibiofilm activity rather than confirmation of clinical safety. Safety evaluation using relevant oral cell models and further assessment of potentially hazardous mineral constituents and silver-ion release will be required before further development of HMNCs for potential oral healthcare applications.

## 5 Conclusion

To the best of our knowledge, this is the first report describing a Kalnar Parpam-derived silver herbomineral nanocomposite and evaluating its antibacterial, antibiofilm, and oxidative-response-associated effects against *S. epidermidis* isolates. The synthesized HMNCs exhibited antibacterial activity at low MIC and MBC concentrations and inhibited both planktonic bacterial growth and biofilm formation under the experimental conditions used in this study. Mechanistic investigations showed that HMNC exposure was associated with extracellular leakage, changes in DCFDA fluorescence, alterations in antioxidant-related parameters, increased MDA-associated signals, and changes in biofilm architecture. These findings provide evidence of membrane-associated, oxidative-response-associated, and biofilm-associated effects; however, the present study does not establish a single causal mechanism of HMNC-mediated antibacterial activity. The findings support further investigation of HMNCs as potential antimicrobial candidates. However, comprehensive cytocompatibility, toxicological evaluation, silver-ion release studies, component-specific controls, and batch-to-batch reproducibility assessment are required before their suitability for oral healthcare applications can be established. Future studies may explore the incorporation of HMNCs into smart oral drug-delivery systems, including stimuli-responsive hydrogels, bioadhesive formulations, nanofibrous scaffolds, and other dental biomaterials. Such approaches may provide opportunities for localized antimicrobial delivery and prolonged interaction with biofilm-associated infections. The potential incorporation of HMNCs into guided tissue regeneration (GTR) or guided bone regeneration (GBR) membranes may also be investigated. However, the possible contribution of mineral constituents such as calcium, magnesium, and silicon to cellular attachment, osteogenic differentiation, or tissue regeneration remains to be experimentally established. Overall, the fabrication of HMNCs represents a Siddha-inspired approach that combines traditional medicinal knowledge with nanomaterial-based antimicrobial strategies. The present findings provide preliminary *in vitro* evidence supporting further investigation of HMNCs for potential oral healthcare applications. Future interdisciplinary research may contribute to the development of novel antimicrobial materials and address challenges associated with biofilm-associated oral infections and antimicrobial resistance, with relevance to SDG 3 (Good Health and Well-Being) and SDG 9 (Industry, Innovation and Infrastructure). Collaboration across traditional medicine, microbiology, nanotechnology, materials science and oral healthcare may further contribute to the broader objectives of SDG 17 (Partnerships for the Goals).

## Data Availability

The datasets presented in this study can be found in online repositories. The names of the repository/repositories and accession number(s) can be found in the article/Supplementary Material.
